# Supplementary material for: Fine needle aspirates characterise the hepatocellular carcinoma immune niche to predict immune checkpoint inhibitor outcomes
Source: JHEP Rep. 2025 Oct 16;8(1):101637. doi: 10.1016/j.jhepr.2025.101637 (PMC12800505; doi:10.1016/j.jhepr.2025.101637)
Supplement: Multimedia component 1 [file mmc1.pdf]

Fine needle aspirates characterise the hepatocellular carcinoma immune niche to predict immune checkpoint inhibitor outcomes

Gloryanne Aidoo-Micah, Stephanie Kucykowicz, Nathalie Schmidt, Vishnu Naidu, Rushabh Shah, Sayani Khara, Tate Mckinnon-Snell, Yiya Zhong, Daniel Brown Romero, Jessica Davies, Laura Pallett, Leo Swadling, Mariana Diniz, Alexa Childs, Upkar Gill, Edward Green, Tim Meyer, Mala Maini

Table of contents

Fig. S1. 2

Fig. S2. 4

Fig. S3 5

Fig. S4 6

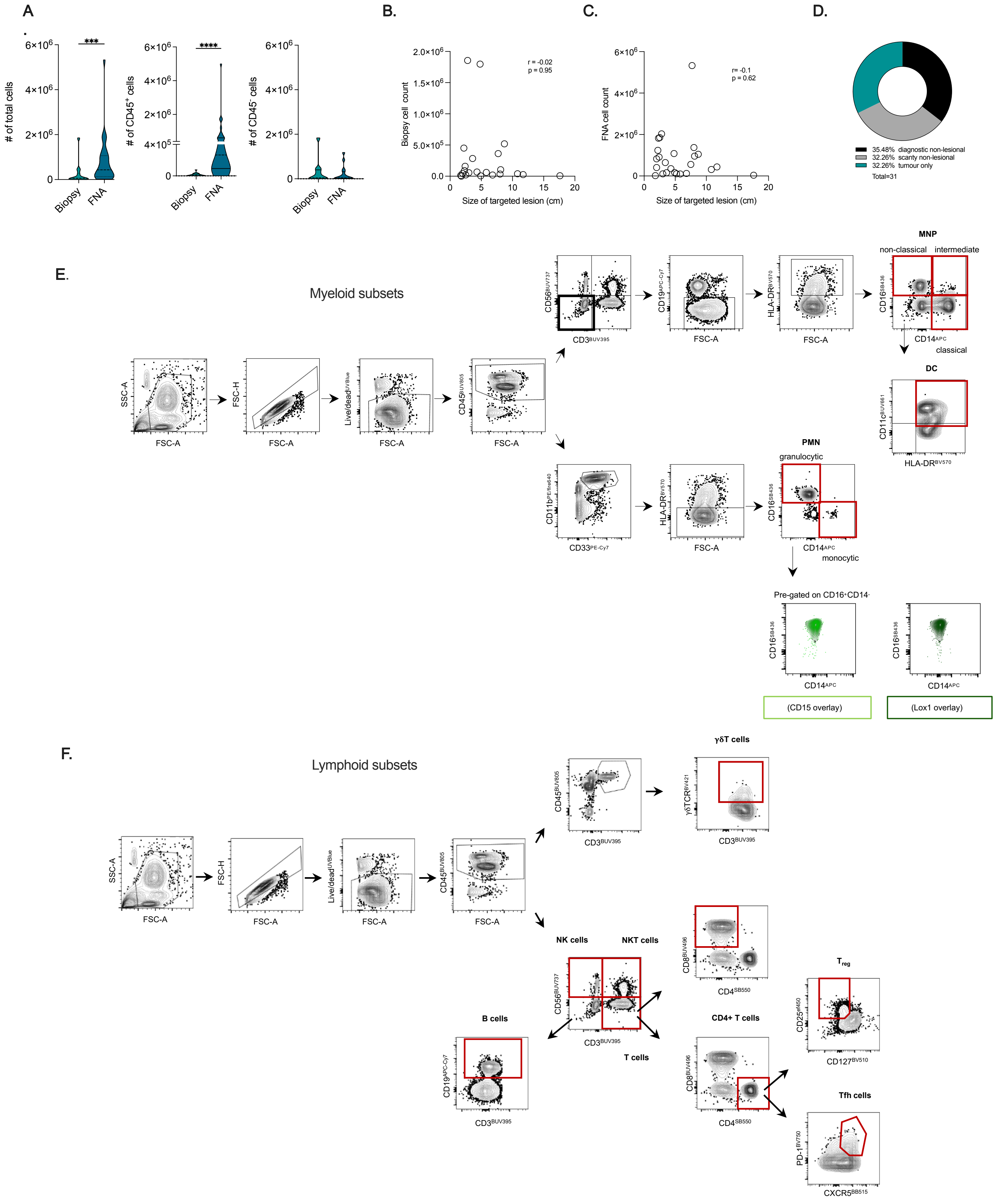

Fig. S1. Identification of immune subsets in HCC. (A) Total number of cells, immune (CD45$^+$) and non-immune (CD45$^-$) obtained from biopsy and FNA, as

quantified by flow cytometric acquisition of whole sample. (b, c) Scatter plots of relationship between the size of HCC and total number of cells from biopsy (B) and FNA (C). (D) Proportion of tumour biopsies containing non-lesional tissue, as reported by routine histological assessment of biopsies from all enrolled patients. (E,F) Representative flow cytometric plots showing sequential manual gating strategy for identification of myeloid (E) and lymphoid (F) subsets. Cells were identified by size (forward scatter area, FSC-A) and granularity (side scatter area; SSC-A), doublet exclusion (forward scatter height; FSC-H), viability staining of live cells and CD45+ immune cells, with subsequent sequential identification of monocytes, myeloid derived suppressor cells (MDSC) and dendritic cells (DC). Overlay of CD15 and Lox1 expression on $CD16^+$ PMN, indicating an overlapping phenotype consistent with suppressive immature PMN (E); and gamma delta (γδ) T cells; $CD56^+CD3^-$ natural killer (NK) cells, $CD56^+CD3^+$ NKT cells, $CD3^+CD56^-$ conventional $CD8^+$T cells, and $CD4^+$T cells, $CD25^+CD127^{lo}$ $CD4^+$ T cells (Treg), $CD19^+$ B cells, and $PD1^+CXCR5^+$T follicular helper (Tfh) cells (F). Significance was determined by non-parametric t-test, Mann-Whitney (A), and nonparametric Spearman correlation (B,C). ***p<0.001,****p<0.0001

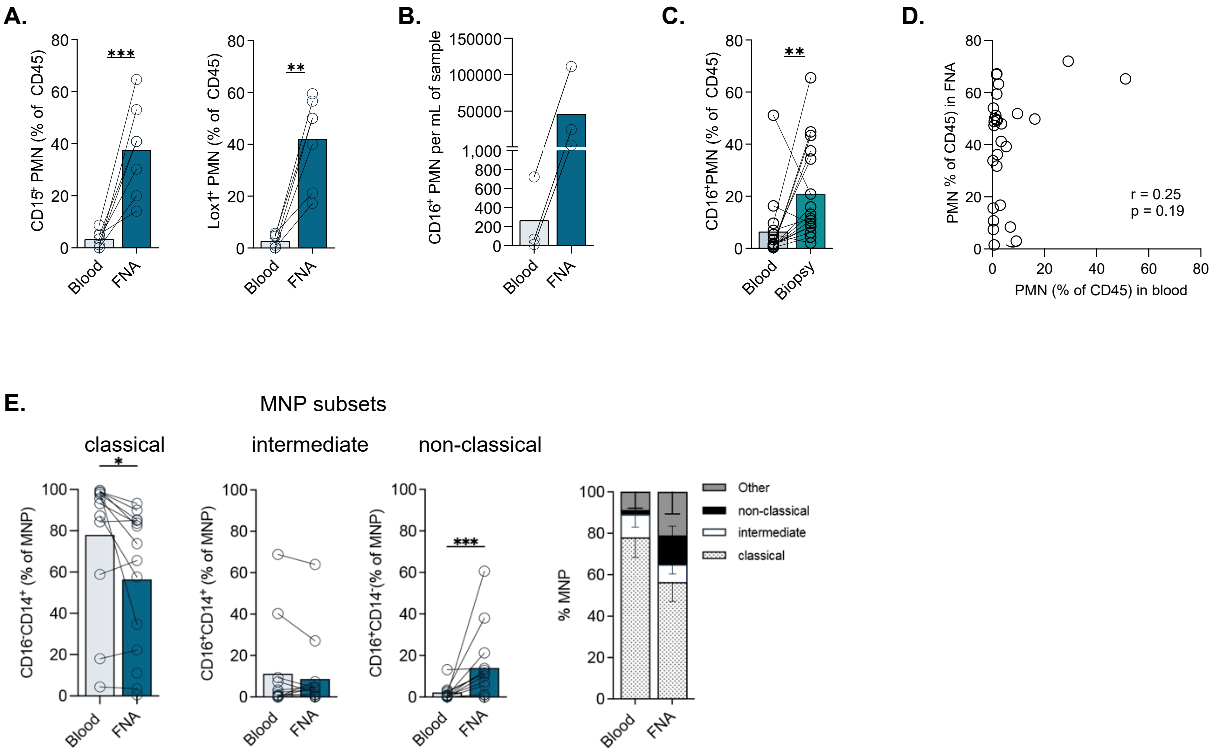

Fig. S2. Differences within the myeloid compartments in blood and tissue. (A) Frequency of immature PMN identified based on expression of CD15 ($CD11b^{+}CD33^{+}HLADR^{-}CD15^{+}CD14^{-}$) or Lox1 ($CD11b^{+}CD33^{+}HLADR^{-}Lox1^{+}CD14^{-}$) between matched blood and FNA in a subset of samples co-stained for these markers (n=7). (B) Absolute counts of $CD16^{+}$ PMN within blood and FNA presented as number of cells per mL of sample using counting beads (n=3). (C) Frequency comparison of $CD16^{+}$ PMN in blood and biopsy. (D) Scatter plot showing correlation between the frequency of $CD16^{+}$ PMN in blood v FNA. (E) Frequency comparison of classical ($CD16^{-}CD14^{+}$), intermediate ($CD16^{+}CD14^{+}$) and non-classical ($CD16^{+}CD14^{-}$) MNP within matched blood and FNA samples (n=11). Data represent mean with significance determined by non-parametric t-test, Wilcoxin test. *$p<0.05$, **$p<0.01$, ***$p<0.001$,****$p<0.0001$ (A-C, E) and nonparametric Spearman correlation (D).

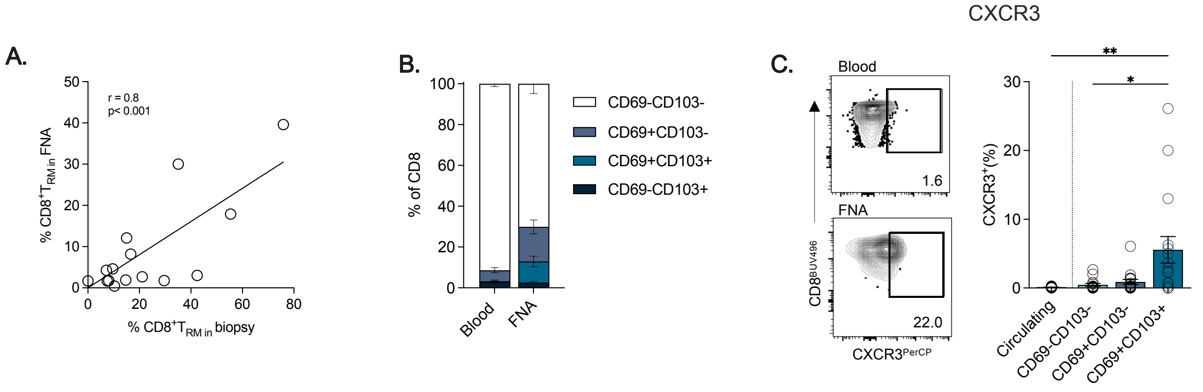

Fig. S3. FNA sampling of tissue compartmentalised immune cells. (A) Scatter plot showing relationship between frequency of $CD8^{+}T_{RM}$ from matched FNA and biopsy(n=15). (B) Summary data of non-resident tumour infiltrating/recirculating ($CD69^{-}CD103^{-}$), single positive $CD69^{+}CD103^{-}$, $CD69^{-}CD103^{+}$ and tissue-resident $CD69^{+}CD103^{+}$ subsets among $CD8^{+}$T cells (n=22). (C) Representative flow cytometric and summary data showing the frequency of $CXCR3^{+}$ cells within $CD8^{+}$T cells circulating in blood, infiltrating ($CD69^{-}CD103^{-}$), $CD69^{+}CD103^{-}$ and tissue-resident $CD69^{+}CD103^{+}CD8^{+}$T cells. Correlation was assessed using Spearman analysis. Data shown are means ± SEM with significance determined by one-way ANOVA with a Dunn's post hoc test for multiple comparisons. *$p<0.05$, **$p<0.01$.

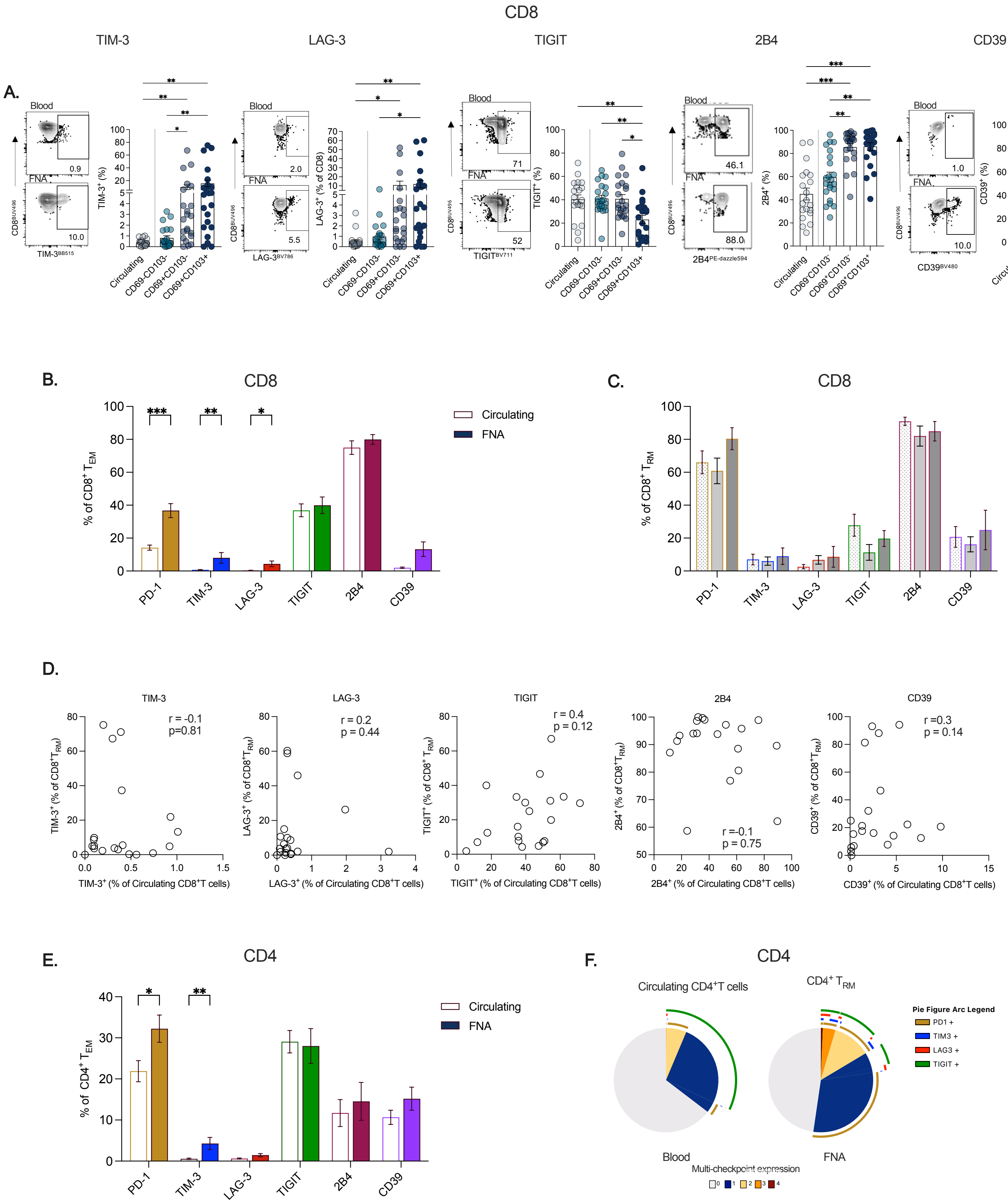

Fig. S4. Enrichment of immune checkpoints in HCC FNA. (A) Representative flow cytometric plots and summary frequency data of CD8^{+}T cells expressing TIM-3, LAG-3, TIGIT, 2B4 and CD39 checkpoint molecules, showing percentage among circulating subsets in blood, tumour recirculating CD69^{-}CD103^{-}, single positive CD69^{+}CD103^{-} or tissue resident CD69^{+}CD103^{+} CD8^{+}T$_{RM}$ in FNA. (B) Summary frequency data of

CD8+TEM (CD62L-) subsets expressing the examined checkpoint molecules in blood and FNA (n=22). (C) Summary frequency data of checkpoint molecule expressing subsets within FNA $CD8^+$TRM grouped by underlying HCC aetiology in samples stained for these markers: viral (HCV/HBV, n=6) and non-viral (ALD, n=5, MASLD, n=7). (D) Scatter plots depicting correlation between the frequency of immune checkpoint expressing $CD8^+$T cells within blood $CD8^+$T cells versus FNA $CD8^+T_{RM}$. (E) Summary frequency data of $CD4^+T_{EM}$ ($CD62L^-$) subsets expressing the examined checkpoint molecules in blood and FNA (n=22). (F) SPICE charts showing the proportion of $CD4^+$T cells expressing PD-1, TIM-3, LAG-3 or TIGIT among global circulating versus FNA $CD69^{hi}CD4^+T_{RM}$. Coloured slices indicate the number of checkpoints expressed on T_{RM}, while colour of the arc indicates the identity of checkpoint molecule expressed (n=16). Data shown are mean ± SEM and significance were determined by one-way ANOVA with a Dunn's post hoc test for multiple comparisons (A), multiple t-test and non-parametric test (B, E) and non-parametric Spearman correlation(D). *$p<0.05$; **$p<0.01$; ***$p<0.001$,****$p<0.0001$.
