# Supplementary material for: Fine needle aspirates characterise the hepatocellular carcinoma immune niche to predict immune checkpoint inhibitor outcomes
Source: JHEP Rep. 2025 Oct 16;8(1):101637. doi: 10.1016/j.jhepr.2025.101637 (PMC12800505; doi:10.1016/j.jhepr.2025.101637)
Supplement: Multimedia component 4 [file mmc4.pdf]

Research article

JHEP|Reports

Fine needle aspirates characterise the hepatocellular carcinoma immune niche to predict immune checkpoint inhibitor outcomes

Authors

Gloryanne Aidoo-Micah, **Stephanie Kucykowicz, Nathalie Schmidt,** …, Edward Green, Tim Meyer, Mala K. Maini[‡]

Correspondence

m.maini@ucl.ac.uk (M.K. Maini), t.meyer@ucl.ac.uk (T. Meyer).

Graphical abstract

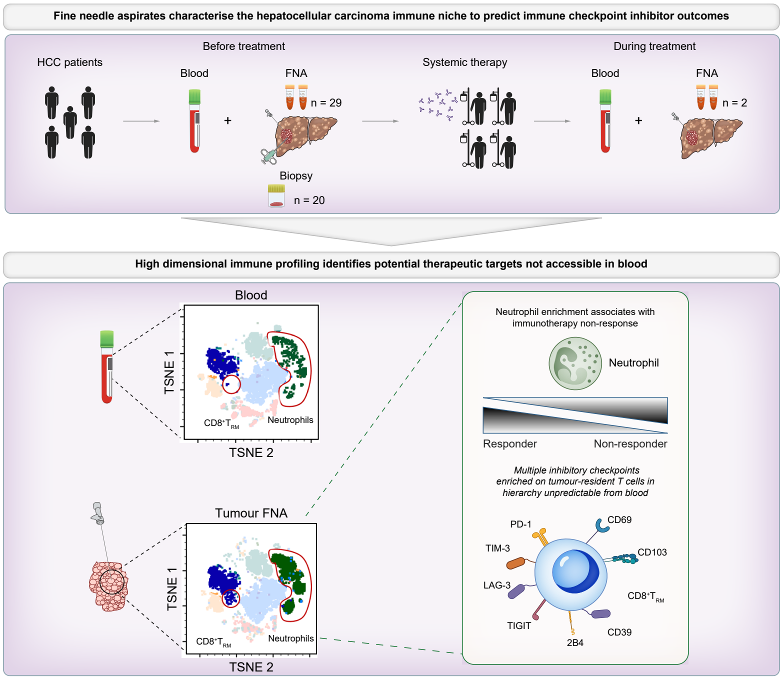

Highlights:

- FNA allow repeat, minimally invasive, comprehensive sampling of the HCC immune niche.
- FNA identify key compartmentalised HCC immunotherapy targets (effectors/suppressors).
- The hierarchy of multiple immune checkpoints enriched on tissue-resident memory T cells is unpredictable from blood T cells.
- Expanded intratumoral neutrophils inversely correlate with key HCC effectors ($CD8^{+}T_{RM}$).
- Expanded intratumoral neutrophils inversely correlate with immunotherapy response.

Impact and implications:

This study addresses the need for simple and minimally invasive assessment of the tumour immune microenvironment in hepatocellular carcinoma, revealing key compartmentalised immunotherapy targets that are not predictable from blood. Findings are important for researchers, clinicians and patients to guide personalised immune checkpoint inhibitor selection and the development of novel approaches to block immunosuppressive neutrophils in order to improve on limited responses to current hepatocellular carcinoma therapies. We demonstrate the potential of rapid fine needle aspirate-based immune profiling to be integrated into larger studies, including clinical trials, to guide personalised selection of patients for existing and future immune checkpoint inhibitors, provide insights into mechanisms of primary and secondary resistance, and inform the development of novel immunotherapy targets.

https://doi.org/10.1016/j.jhepr.2025.101637
© 2025 The Authors. Published by Elsevier B.V. on behalf of European Association for the Study of the Liver (EASL). This is an open access article under the CC BY license (http://creativecommons.org/licenses/by/4.0/). JHEP Reports, 2026, 8, 1–13

Fine needle aspirates characterise the hepatocellular carcinoma immune niche to predict immune checkpoint inhibitor outcomes

Gloryanne Aidoo-Micah[1,4], **Stephanie Kucykowicz**[1,†], **Nathalie Schmidt**[1,†], Vishnu Naidu[2], Rushabh Shah[2], Sayani Khara[2], Tate Mckinnon-Snell[1], Yiya Zhong[1], Daniel Brown Romero[1], Jessica Davies[1], Laura Pallett[1], Leo Swadling[1], Mariana Diniz[1], Alexa Childs[4], Upkar Gill[3], Edward Green[2], Tim Meyer[4,*,‡], Mala K. Maini[1,‡,*]

JHEP Reports **2026**. vol. 8 | 1–13

Check for updates

Background & Aims: Antitumour immunity involves a complex balance of immune effectors and regulators, many of which are adapted or compartmentalised within the local microenvironment. How this influences responses to immunotherapy in hepatocellular carcinoma (HCC) is poorly understood because of limited access to the tumour immune landscape. We postulated that fine needle aspirates (FNA) could allow for minimally invasive, in-depth tumour immune profiling in patients with advanced HCC.

Methods: Patients with radiological evidence of advanced HCC were prospectively enrolled to provide matched blood, FNA (n = 29 baseline, 2 also on treatment) and biopsy (n = 20 baseline only) for *ex vivo* spectral flow cytometric characterisation of multiple immune populations.

Results: FNA yielded more viable leukocytes than biopsies (mean 800,000 *vs*. 250,000 cells) allowing reproducible characterisation of a broad range of viable immune effectors and regulators enriched within the tumour. Tissue-resident memory $CD8^+T$ cells ($CD8^+T_{RM}$), a subset critical to cancer control that are excluded from blood, could also be aspirated from HCC for phenotypic/functional assessment (mean 10% *vs.* 0.1%, p <0.0001). PD-1 and alternative checkpoints (TIM-3/LAG-3/2B4/CD39) were strikingly enriched on $CD8^+T_{RM}$ and $CD4^+T_{RM}$, which were also more likely to co-express multiple checkpoints than their circulating counterparts ($CD8^+T_{RM}$: 21.6% *vs.* 7.4%, p = 0.0003, $CD4^+T_{RM}$: 16.4% *vs.* 5.6%, p = 0.011). Expression of checkpoints on circulating T cells was discordant with levels on the fraction compartmentalised within tumours. FNA revealed an intratumoral expansion of neutrophils with an immunosuppressive phenotype that were increased in non-responders to immunotherapy (mean 50.2% *vs.* 25.5% in responders, p = 0.015) and correlated inversely with $CD8^+T_{RM}$ and $CD4^+T_{RM}$ frequencies (r = 0.6, p = 0.001).

Conclusion: FNA are suitable for rapid, comprehensive sampling of HCC prior to and during immunotherapy, revealing features of the tissue-resident tumour immune niche that cannot be predicted from blood. These features have the capacity to predict clinical outcomes.

© 2025 The Authors. Published by Elsevier B.V. on behalf of European Association for the Study of the Liver (EASL). This is an open access article under the CC BY license (http://creativecommons.org/licenses/by/4.0/).

Introduction

Primary liver cancer is a significant global health challenge, representing the third leading cause of cancer-related deaths.[1] Previous incidence estimates of over 800,000 cases per year are projected to exceed a million affected annually in 2025.[1,2] Hepatocellular carcinoma (HCC) accounts for over 90% of cases and typically develops in the setting of chronic liver inflammation or cirrhosis. Late presentation and high rates of recurrence or progression after surgical resection or locoregional therapy result in a high proportion of patients requiring systemic therapy. Recent trials have demonstrated superior outcomes for immune checkpoint inhibitor (ICI)-based therapy, which has replaced tyrosine kinase inhibitors (TKIs) as the recommended first-line treatment for advanced HCC.[3–5] Despite this progress, response rates are 30% or less and 4-year survival is 25% at best,[3,6] highlighting the urgent need for biomarkers to select the subset of patients most likely to respond to current immunotherapy. The emerging availability of alternative ICI regimens for HCC underscores the need for accurate assessment of their hierarchical expression.[7,8] Further in-depth immune profiling of HCC is also required to discover alternative immunosuppressive mechanisms that could be targeted by novel therapeutic approaches.

* Corresponding authors: Prof Mala K Maini, Institute of Immunity and Transplantation, Pears Building, Rowland Hill St, London NW3 2PP, United Kingdom and Prof Tim Meyer, UCL Cancer Institute, Huntley Street, London, WC1E 6BT, United Kingdom.
E-mail addresses: m.maini@ucl.ac.uk (M.K. Maini), t.meyer@ucl.ac.uk (T. Meyer).
† Joint second authors
‡ Joint senior and corresponding authors
https://doi.org/10.1016/j.jhepr.2025.101637

Tumours comprise highly immunosuppressive niches that are challenging to overcome with immunotherapy; in the case of HCC this is exacerbated by the capacity of liver tumours to co-opt the inherently tolerogenic hepatic environment, further complicated by underlying disease aetiology and resultant background inflammation and fibrosis.[8–10] The heterogeneity of this landscape is only beginning to be dissected.[11–14] While analysis of peripheral blood can provide insights into tumour immune responses,[15] many immune effectors and regulators involved in disease control or progression are altered, enriched or completely compartmentalised at the site of disease.[16] In particular, tissue-resident T cells (T_{RM}) have been shown to represent critical effectors for immune control of many tumours including HCC.[10,13,17,18] This sequestration restricts their representation in peripheral blood and highlights the importance of studying the local tissue microenvironment to identify reliable immunotherapy biomarkers and therapeutic targets.

Unlike most solid tumours, HCC has commonly been diagnosed using imaging criteria alone in patients with cirrhosis,[2,19] although biopsy is increasingly recommended for histological confirmation before embarking on systemic treatment.[19,20] HCC biopsies, however, are not ideal for flow cytometric analysis of intratumoral immune cells since they often include some adjacent unaffected liver tissue[14] and require tissue digestion. We and others have previously used a fine bore (22-25 gauge) needle for ultrasound-guided aspiration of immune cells from the liver, demonstrating that these samples capture a representative fraction of the immune landscape and allow for longitudinal assessment because of their minimally invasive nature.[21–26] These fine needle aspirates (FNA) generate a cell suspension not requiring any tissue processing, suitable for phenotypic and functional analysis by flow cytometry or RNA sequencing immediately or following cryopreservation.[21,27] FNA have previously been safely applied to the cytological assessment of HCC[28,29] but have not been tested for their capacity to sample immune cells in this setting. Here we present the first study analysing the capacity of minimally invasive FNA[23–26] to thoroughly sample the tumour-sequestered immune landscape of HCC in patients eligible for immunotherapy. Our findings demonstrate the potential of this rapid *ex vivo* profiling approach to guide the personalised selection of patients for existing and future checkpoint inhibitors, reveal mechanisms of primary and secondary resistance, and identify novel immunotherapy targets.

Patients and methods

Patient cohort

Participants were undergoing percutaneous biopsy of radiologically diagnosed liver lesions.[19] Thirty-one patients were included in the study. Twenty-nine patients had matched blood, FNA and biopsy, while two had blood and clinically indicated biopsy samples only, as pre-defined eligibility criteria for research FNA sampling were not met. In these cases, one biopsy core surplus to diagnostic requirements was processed for research purposes.

Thirty patients had cirrhosis. The underlying aetiology was viral in six cases (HBV n = 1; HCV n = 4; HBV-HCV coinfection n = 1).

Of the remaining 24 patients, 19 had a single non-viral diagnosis comprising metabolic dysfunction-associated steatotic liver disease (MASLD n = 7), alcohol-related liver disease (ALD n = 10, of whom 8 had FNA), or haemochromatosis (n = 2). Three patients had ≥2 non-viral diagnoses, and two had no documented cause.

Additional cohort and clinical characteristics are summarised in Table 1.

Ethics approval

This study was approved by the West Midlands-Solihull Research Ethics Committee (REC reference 21/WM/0205) and complied with the Declaration of Helsinki. All participants gave written consent prior to inclusion in the study and storage of all samples complied with the Human Tissue Act.

Key eligibility criteria

Participants were adults (≥18 years) with unresectable HCC diagnosed by EASL (European Association for the Study of the Liver) radiological criteria or histology, classified as BCLC (Barcelona Clinic Liver Cancer) stage B or C, and with an ECOG (Eastern Cooperative Oncology Group) performance status of 0-2 prior to initiation of systemic treatment. Additional inclusion criteria were a platelet count of $\geq 50 \times 10^9$/L, international normalized ratio ≤1.2, Child-Pugh class A liver function, and the ability to safely pause antiplatelet or anticoagulant therapy.

Table 1. Study participant characteristics.

Patient characteristics (n = 31)	
Age (years, IQR)	71 (66-77)
Sex, male (n,%)	24 (77)
Underlying liver disease[1] (n,%)	
Viral	
HBV infection	2 (6)
HCV infection	5 (16)
Non-viral	
ALD	14 (45)
MASLD	9 (23)
Other	3 (9)
Unknown	2 (6)
ECOG performance status (n,%)	
0	9 (29)
1	21 (68)
2	1 (3)
Cirrhosis present (n,%)	30 (97)
Mode of diagnosis	
Imaging	2 (7)
Histology	29 (93)
Number of lesions	
1	5 (16)
2	5 (16)
3 or more	21 (68)
Size of lesion sampled[2] (median, range) (cm)	4.6 (1.7 – 18)
Macrovascular invasion (n,%)	11 (35)
Extrahepatic spread (n,%)	5 (16)
Tumour grade (29) (n,%)	
Well differentiated	5 (17)
Moderately differentiated	20 (69)
Poorly differentiated	4 (14)

ALD, alcohol-related liver disease; ECOG, Eastern Cooperative Oncology Group; MASLD, metabolic dysfunction-associated steatotic liver disease.
[1]Total exceeds number of patients due to overlapping aetiologies.
[2]Total of four lesions were not measurable – consisting of portal vein thrombus n = 2, or infiltrative diffuse lesions n = 2.

Sample collection

Matched blood and tissue samples were collected from patients prior to initiation of treatment for HCC. Where feasible, additional blood and FNA were obtained within 3 months of starting therapy.

Blood

Peripheral blood was collected via venepuncture using standard aseptic techniques. A maximum of 50 ml of whole blood was drawn into lithium heparin-coated tubes and processed within 2 h.

FNA and biopsy

Local anaesthesia (1% lidocaine or combination of 1% lidocaine and 0.5% bupivacaine) was used in the subcutaneous plane and infiltrated to the liver capsule under ultrasound guidance. A 22-gauge spinal needle with an internal trocar was inserted along the anaesthetised tract to the liver capsule edge and inserted into the liver parenchyma and into the lesion. The internal trocar was then removed and attached to a 20 ml Luer lock syringe filled with 5 ml of RPMI-1640 medium (Sigma-Aldrich) via connecting tubing. Under continuous negative pressure, the needle was inserted and withdrawn whilst remaining in the lesion, with a fanning technique as previously described.[21] The needle was then removed from the patient and the pre-existing media was flushed through into a falcon tube. Additional flushes of the syringe with RPMI-1640 were performed to ensure maximal cell yield. The FNA was repeated a total of two times using a new 22-gauge needle for the second pass. Following this, two percutaneous biopsies were performed using an 18-gauge BioPince Ultra Full Core Biopsy Instrument (Argon Medical Devices) via a 17-gauge co-axial needle to minimise the number of capsular punctures. One tissue core was used for histopathological diagnosis and the other for research.

PBMC and TIL isolation

PBMCs were isolated from heparinised whole blood via density gradient centrifugation using Pancoll (Pan Biotech). Tumour-infiltrating lymphocytes (TILs) from FNA were obtained by centrifugation of the aspirated cell suspension to pellet cells, followed by red cell lysis (Biolegend). Additional lysis steps were performed for samples with visible blood contamination, adapting previously described protocols.[21] TILs from biopsies were isolated by mechanical dissociation as previously described for the extraction of leukocytes from liver biopsy tissue.[32] Briefly, tissue was gently dissociated into small fragments using a cell scraper and passed through a 70 μm filter (Greiner) to remove debris. Freshly isolated cells were seeded in a 96-well plate at a maximum density of $1x10^6$ cells per well for direct *ex vivo* staining.

Flow cytometry staining

Multiparametric spectral flow cytometry was used for phenotypic and functional analysis of PBMCs and TILs. Cells were stained with a fixable live/dead dye (Invitrogen) before incubation with saturating concentrations of surface monoclonal antibodies (mAbs) diluted in a 1:1 solution of PBS (Invitrogen) and Brilliant Violet Buffer (BD Biosciences). For surface marker characterisation, cells were fixed with Cytofix (BD Biosciences). For intracellular staining, cells were fixed and permeabilised with Cytofix/Cytoperm (BD Biosciences), and subsequently incubated with saturating concentrations of mAbs diluted in 0.1% saponin (Sigma-Aldrich) for 30 min at 4 °C. Full details on fluorescent mAbs are provided in Table S1.

All samples were acquired on a Cytek® Aurora spectral cytometer (Cytek) and analysed using FlowJo software, version 10.0.8r1 (BD). Two partially overlapping 36-colour panels were used interchangeably for immune subset identification and T-cell profiling. An additional panel was used to assess intracellular cytokine staining in a subset of FNA samples. Samples yielding fewer than 5,000 events following doublet exclusion were excluded from downstream analysis. The number of samples included in specific analyses are indicated in the respective figure legends.

Functional assessment of FNA-derived TILs

A total of 0.5×10^6 TILs from FNA were cultured overnight at 37 °C in a humidified atmosphere with 5% CO_2 in complete RPMI supplemented with 20 IU/ml recombinant human IL-2 (PeproTech), in the presence of 1 μg/ml brefeldin A (Sigma-Aldrich) and BD GolgiStop™ (BD Biosciences). Cells were either unstimulated as matched controls or stimulated with 0.5 μg/ml immobilised anti-CD3 and 0.5 μg/ml soluble anti-CD28 antibodies (eBioscience). Functionality of $CD4^+$ and $CD8^+$T cells was assessed by intracellular cytokine staining, as detailed in the ‘Flow cytometry’ section above.

Statistical analysis

Statistical analyses were performed in Prism 9.0 (GraphPad) using appropriate methods as indicated in each figure legend (Wilcoxon signed-rank *t* test, Mann-Whitney *U* test, Friedman test (ANOVA) with a Dunn’s *post hoc* test for pairwise multiple comparisons between each group, or Spearman correlation). Where significant, the differences were marked on the appropriate figures. All tests were performed as two-tailed tests, and for all tests, significance levels were defined as not significant $p \geq 0.05$; *p <0.05; **p <0.01; ***p <0.001; ****p <0.0001.

Results

FNA samples a comprehensive range of viable myeloid and lymphoid cells from the HCC niche

Thirty-one patients diagnosed with unresectable HCC consented to provide matched blood, core biopsy and FNA at the time of routine diagnostic biopsy, prior to commencing systemic treatment (Fig.1A, Table 1 and Methods for patient characteristics). Two FNA passes were obtained using ultrasound guidance and the ‘fanning technique’ to obtain wide coverage of the tumour, whilst avoiding unaffected liver (Methods).[21,30,31] The FNA was spun down for immediate multiparameter antibody staining as previously described, with additional lysis steps to remove red blood cells that commonly contaminated samples because of the highly vascular nature of HCC. Leukocytes obtained from density centrifugation of PBMCs and from mechanical digestion of surplus biopsy tissue (as previously described) were stained in parallel.[21,32]

Direct *ex vivo* flow cytometry of the whole sample revealed that FNA yielded significantly more total cells than the more

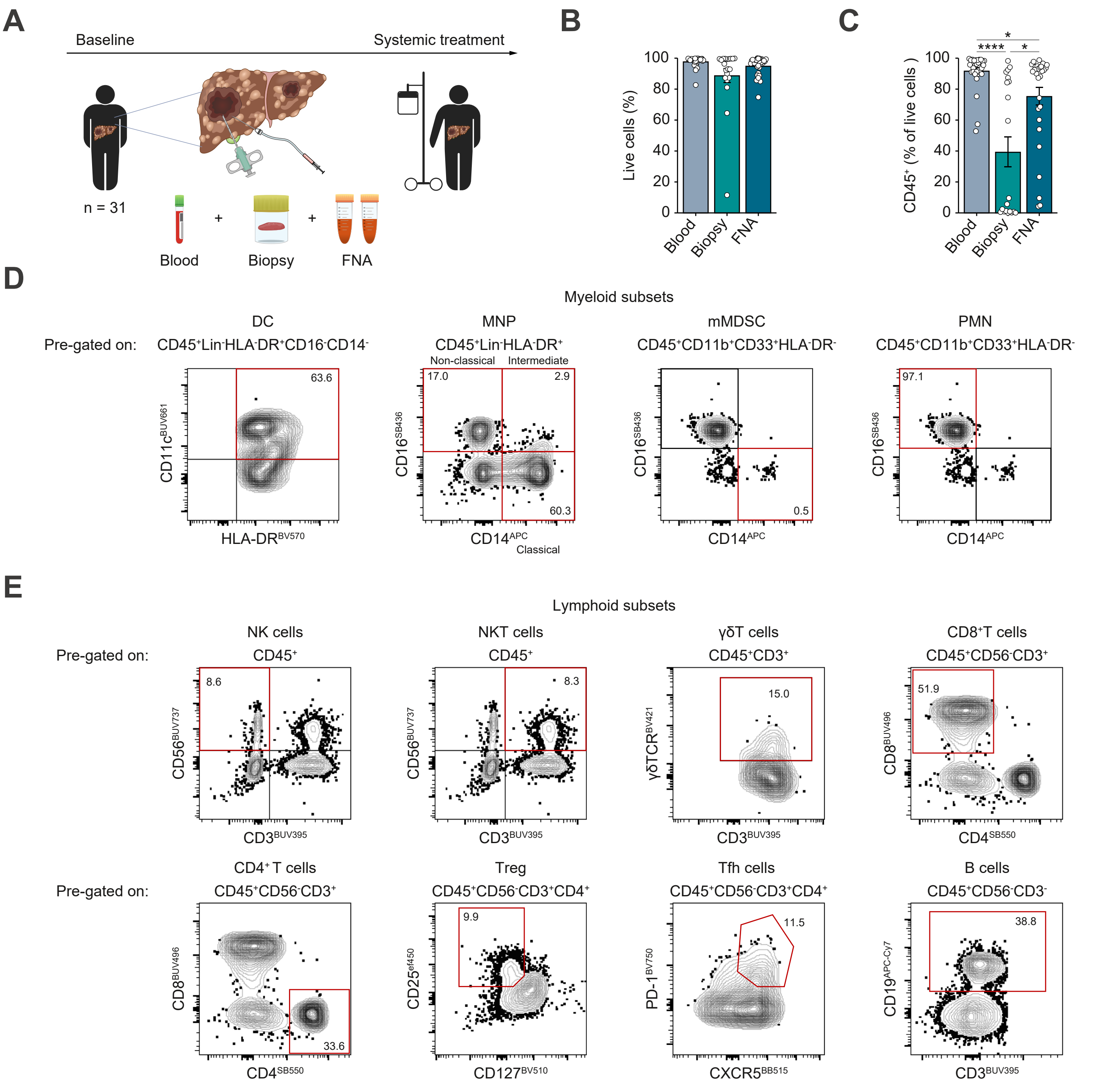

Fig. 1. FNA samples diverse immune populations in HCC tumours. (A) Schema of study outline. Blood, biopsy and FNA samples were obtained from 31 patients with radiological evidence of unresectable HCC prior to systemic therapy. PBMCs (n = 31) and TILs from biopsy (n = 31) or FNA (n = 29) were analysed directly *ex vivo* by multiparametric spectral flow cytometry. Eleven biopsies yielded insufficient events for reliable cytometric characterisation. (B) Percentage of live cells within PBMCs (n = 31) and TILs from biopsy (n = 20) and FNA (n = 29). (C) Frequency of $CD45^+$ cells as a percentage of live cells, pre-gated by forward and side scatter area, doublet exclusion, live cells. (D, E) Representative flow cytometric plots of myeloid subsets (D) and lymphoid subsets (E) identified within FNA. Data represent mean ± SEM. Significance was determined by non-parametric *t* test, Mann-Whitney (B), and one-way ANOVA, Kruskal-Wallis test (C). *p <0.05, ****p <0.0001. FNA, fine needle aspiration; HCC, hepatocellular carcinoma; PBMC, peripheral blood mononuclear cell; TIL, tumour-infiltrating lymphocyte.

invasive needle biopsies (mean 800,000 vs. 250,000 cells), with the number of cells recovered from either approach being independent of the size of the HCC lesion (Fig. S1A–C). A high proportion of the cells obtained from both FNA and biopsies were viable, comparable to blood (Fig. 1B). However, FNA acquired a significantly higher proportion of leukocytes (marked by CD45) than biopsies (Fig. 1C); after adjusting for total sample cell counts, this constituted a selective absolute increase in leukocytes (rather than non-CD45 populations) obtained by FNA (Fig. S1A). The low proportion (mean 39% of live cells) and absolute number of leukocytes isolated from many biopsy samples, together with histologically reported contamination of biopsies with non-lesional tissue (utilised for diagnosis of underlying liver disease) (Fig. S1D), limited

consistent immune subset characterisation in these samples; subsequent tumour immune profiling was therefore focused on FNA.

Phenotypic characterisation using a 36-colour spectral cytometry panel allowed comprehensive immune profiling, revealing that FNA TILs contained a diverse array of immune cells (Fig. 1D,E and S1E,F). Within the myeloid compartment of FNA, we identified cell types involved in antigen presentation and T-cell regulation (Fig. 1D). These included dendritic cells (DCs; HLA-DR$^+$CD14$^-$CD16$^-$CD11c$^+$ within the lineage (CD3,CD56,CD19) negative gate), non-classical (HLA-DR$^+$CD14$^-$CD16$^+$), intermediate (HLA-DR$^+$CD14$^+$CD16$^+$) and classical (HLA-DR$^+$CD14$^+$ CD16$^-$) mononuclear phagocytes (MNPs), the latter of which have been associated with the generation of tumour-associated macrophages within the tumour niche.[33] Immature myeloid cells with a phenotype previously linked with monocytic myeloid-derived suppressor cells (mMDSCs) (CD11b$^+$CD33$^+$HLA-DR$^-$CD16$^-$CD14$^+$) and polymorphonuclear neutrophils (PMNs) with the phenotype used to identify suppressive granulocytic MDSCs (gMDSCs, also known as tumour-associated neutrophils [TANs]) (CD11b$^+$CD33$^+$HLA-DR$^-$CD14$^-$CD16$^+$/CD15$^+$/Lox-1$^+$) were also aspirated from HCC (Fig. 1D).

Within the lymphoid compartment of FNA-derived TILs, we also observed a broad range of cell types including antitumour effector subsets such as natural killer (NK) cells (CD56$^+$CD3$^-$), NKT cells (CD56$^+$ CD3$^+$), $\gamma\delta$T cells (CD3$^+\gamma\delta$TCR$^+$), CD8$^+$T cells (CD3$^+$CD56$^-$CD8$^+$) and CD4$^+$T cells (CD3$^+$CD56$^-$CD4$^+$). Other subsets with antitumour and/or regulatory potential identified included regulatory T cells (Tregs) (CD4$^+$CD25$^+$CD127^{lo}), follicular helper T (Tfh) cells (CD4$^+$CXCR5$^+$PD1$^+$) and B cells (CD3$^-$CD56$^-$CD19$^+$) (Fig. 1E). Thus, FNA comprehensively sampled tumour immune cells that could be further characterised to dissect antitumour and tumour-promoting features in HCC.

Distinct immune landscapes in FNA and blood samples

Next, the proportions of major immune subsets (as a percentage of CD45$^+$ leukocytes) were compared between paired blood and FNA. T-distributed stochastic neighbour embedding (t-SNE) allowed 2-D visualisation of their unique immune composition, with cell types clustered based on similarly expressed markers following dimensionality reduction applied to a subset of nine representative samples (Fig. 2A). Relative to blood, FNA contained a significant expansion of neutrophils with a phenotype previously associated with potent suppressive capacity, including when isolated from human liver (CD11b$^+$CD33$^+$HLA-DR$^-$ with co-expression of CD16/CD15/ Lox1) (Fig. 2A and S2A).[34,35] This expansion of PMNs in FNA was confirmed in the summary data of all patients following manual gating, as a percentage of CD45$^+$ cells retrieved (mean 5.8% blood *vs*. 41% FNA) (Fig. 2B and S2A) and by absolute cell numbers (calculated in a small subset of samples, Fig. S2B). An equivalent expansion of this subset over percentages in blood was seen in the 16 HCC biopsies with sufficient cells for analysis (Fig. S2C). Of note, there was striking variability in the frequency of this population of neutrophils between different HCC FNA, with one group clustering around a mean of 9% and another around 50%, and no correlation with their frequencies in blood (Fig. 2B and S2D). Conversely, the frequencies of mMDSCs, MNPs and DCs were all reduced within FNA TILs relative to PBMCs (Fig. 2B). Within the MNP population, the classical subset decreased, intermediate remained unchanged, whereas non-classical were increased (Fig. S2E).

The lymphocyte compartment was proportionally decreased within FNA, in line with the relative myeloid expansion, with significantly lower percentages of T cells, NK cells and B cells, although NKT cell frequencies were maintained (Fig. 2C). There were differences within the T-cell compartment in HCC sampled by FNA, with a higher proportion of CD8$^+$T cells and less CD4$^+$T cells compared to the peripheral blood (Fig. 2D).[21,36,37] Like CD8$^+$T cells, $\gamma\delta$T cells with potential for antitumour function[36] were also relatively enriched within the CD3$^+$ fraction of FNA (Fig. 2D). By contrast, the proportion of CD4$^+$T cells with a surface phenotype suggestive of regulatory function (Treg) or of circulating Tfh cells was not significantly different in FNA compared to matched blood (Fig. 2E).

Taken together, FNA sampled a notably increased proportion of neutrophils at the expense of other subsets (T cells, NK cells, B cells, mMDSCs, MNPs, DCs), and revealed a selective expansion of CD8$^+$ and $\gamma\delta$T cells (but not CD4$^+$T cell subsets), within tumour leukocytes compared to matched blood samples (Fig. 2C-F). These findings were preserved among HCC of different aetiologies (n = 21 that could be classified into single aetiolgies: viral n = 6; ALD n = 8 and MASLD n = 7; Fig. 2G).

FNA retrieves tissue-resident lymphocytes compartmentalised within HCC

To assess the capacity of FNA to extract cells from liver tumours that are sequestered out of the blood, we looked for the presence of T_{RM}, a population known to provide critical local cancer surveillance.[17,37] We and others have previously shown that human liver CD8$^+$T$_{RM}$, characterised by potent effector function and long-lived hepatic progeny, can be defined by their co-expression of the retention markers CD69 and integrin αEβ7 CD103.[16,18,38] Despite their adaptations for tight retention within tissues, we were able to aspirate CD8$^+$T$_{RM}$ from HCC, with a highly variable frequency (mean 10%, range 0.4-40% CD69$^+$CD103$^+$ of total CD8$^+$T cells, Fig. 3A) (that strongly correlated with frequencies obtained from core biopsies, Fig. S3A). As expected, CD69$^+$CD103$^+$CD8$^+$T$_{RM}$ frequencies were negligible in matched blood (mean 0.2%, Fig. 3A), confirming that they retained tissue compartmentalisation in patients with HCC. CD69$^+$CD103$^-$CD8$^+$T cells ('single-positive') were also enriched in HCC FNA compared to blood, some of which may also constitute T_{RM} but can be difficult to distinguish from their circulating activated counterparts (Fig. S3B). To further identify bona fide CD8$^+$T$_{RM}$, we investigated the expression of additional homing and retention markers. The collagen-binding integrin CD49a, reported as a T_{RM} marker in other human tissues[39,40] and on human liver $\alpha\beta$ and $\gamma\delta$T cells,[36,41] showed significantly enriched expression on the CD69$^+$CD103$^+$CD8$^+$T$_{RM}$ fraction of FNA compared to either circulating CD8$^+$T cells or tumour-recirculating (CD69$^-$ CD103$^-$CD8$^+$) T cells, consistent with T_{RM} status (Fig. 3B). CD49a was also increased on single-positive CD69$^+$CD103$^-$ CD8$^+$T cells compared to circulating CD8$^+$T cells (Fig. 3B). The tissue-homing chemokine receptor CXCR6 (binding to CXCL16 which is expressed in HCC[42,43]) was more highly expressed on CD69$^+$CD103$^+$CD8$^+$T$_{RM}$ and CD69$^+$CD103$^-$

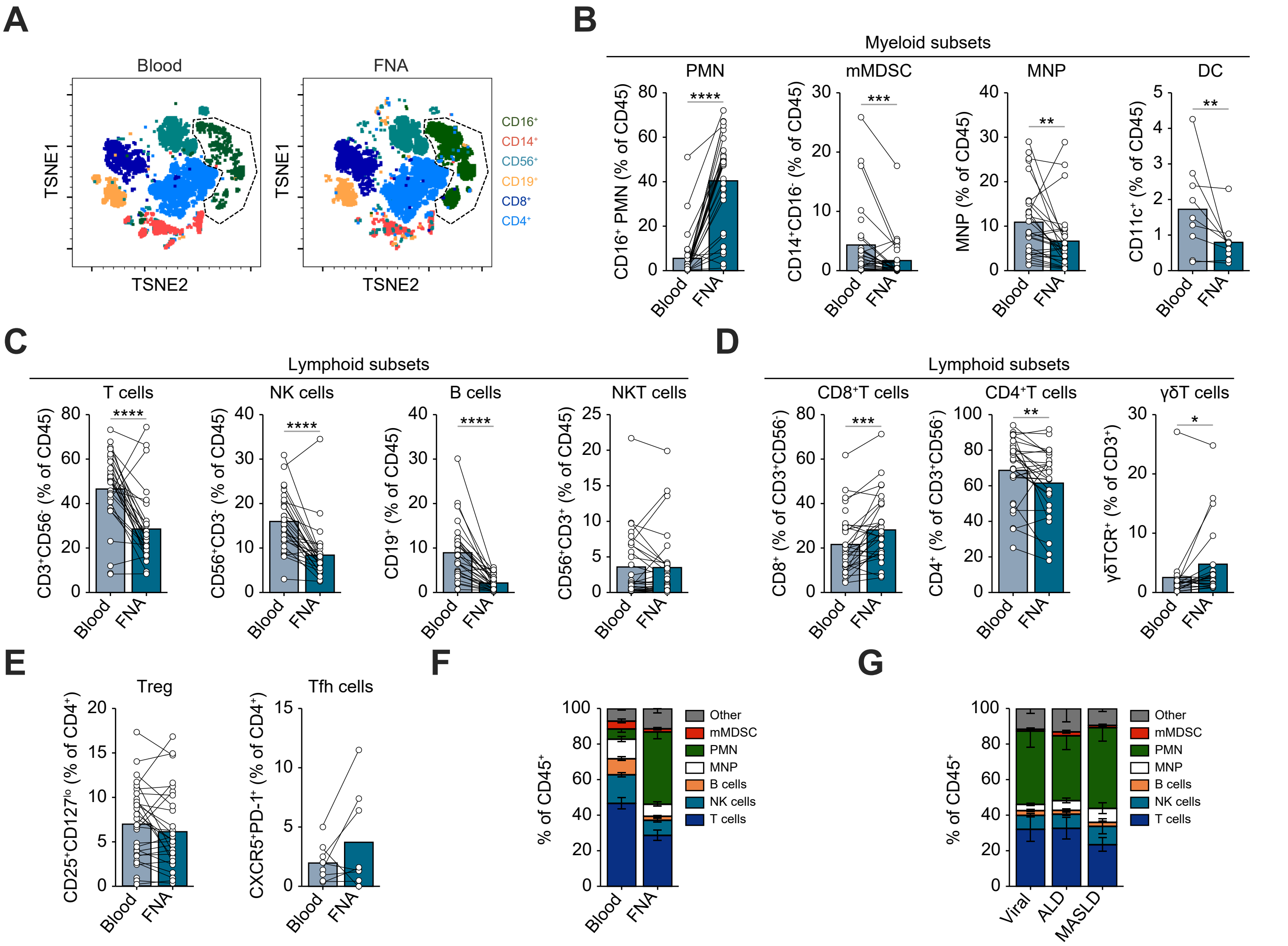

Fig. 2. Distinctions between immune landscapes in blood and FNA. (A) t-SNE representation of live $CD45^+$ leukocytes from blood and FNA (n = 9), clustered by flow cytometric marker expression. Dominant immune cell lineages are indicated. (B) Frequency comparison between matched blood and FNA of neutrophils (PMNs) ($CD11b^+CD33^+HLA\text{-}DR^-CD16^+$) (n = 29), mMDSCs ($CD11b^+CD33^+HLA\text{-}DR^-CD14^+$) (n = 29), total MNPs (including classical ($CD16^-CD14^+$), intermediate ($CD16^+CD14^+$), non-classical ($CD16^+CD14^-$) pre-gated on lineage (CD3,CD56,CD19; $Lin^-HLA\text{-}DR^+$) (n = 29) and DCs ($CD11c^+$) (n = 9). (C) Frequency comparison of lymphoid subsets including T cells ($CD3^+CD56^-$) (n = 29), NK cells ($CD56^+CD3^-$) (n = 29), B cells ($CD3^-CD56^-CD19^+$) (n = 29) and NKT cells ($CD56^+CD3^+$) (n = 29) between blood *vs.* FNA. (D) Comparison of $CD8^+$T, $CD4^+$T (n = 29) and γδT cell frequencies (n = 9) within the T-cell compartment in matched blood and FNA. (E) Comparison of $CD4^+$T-cell subset frequencies of Tregs ($CD25^+CD127^{lo}$) (n = 29) and Tfh cells ($CXCR5^+PD1^+$) (n = 8) in blood and FNA. (F) Summary data of major myeloid and lymphoid subsets as a proportion of CD45 within blood and FNA (n = 29). (G) Proportions of $CD45^+$ subsets separated by underlying diagnosis of viral (HCV or HBV infection) n = 6, ALD n = 8 or MASLD n = 7, where a single aetiology was found. Data represent mean ± SEM; significance was determined by non-parametric *t* test, Wilcoxin test. **p* <0.05, ***p* <0.01, ****p* <0.001, *****p* <0.0001. ALD, alcohol-associated liver disease; DC, dendritic cell; FNA, fine needle aspiration; MNP, mononuclear phagocyte; mMDSC, monocytic myeloid-derived suppressor cell; MASLD, metabolic dysfunction-associated steatotic liver disease; NK cell, natural killer cell; NKT cell, natural killer T cell; PBMC, peripheral blood mononuclear cell; PMN, polymorphonuclear cell; Tfh, follicular helper T; Treg, regulatory T cell; γδT cell, gamma delta T cell; t-SNE, t-distributed stochastic neighbour embedding.

$CD8^+$T cells within FNA than circulating and tumour-recirculating $CD8^+$T cells, with CXCR3 (binding to CXCL9,10,11) showing a similar trend for enrichment (Fig. 3C and S3C).

We have also previously demonstrated that a liver-resident population of $CD4^+$T cells, distinguished by high expression of CD69 ($CD69^{hi}CD4^+$T cells), has potent capacity for cytokine production, is excluded from the circulation and can persist long-term.[44] This is distinct from a subset with intermediate CD69 expression ($CD69^{int}CD4^+$T cells) that can include activated $CD4^+$T cells able to recirculate.[44] We found that $CD69^{hi}CD4^+$T cells could be aspirated from the majority of tumours sampled, representing the first demonstration that $CD4^+T_{RM}$ can populate human HCC. The frequency of $CD69^{hi}CD4^+T_{RM}$ in FNA ranged from 0.2 to 36% (mean 9.1%), significantly greater than in matched blood samples (mean 1%, Fig. 3D). As previously described for $CD69^{hi}CD4^+T_{RM}$ in the liver, this population in HCC FNA were enriched for CD49a and CXCR6 compared to peripheral and tumour-recirculating $CD4^+$T cells (Fig. 3E,F).

To examine the utility of FNA for evaluating functionality of intratumoral T cells, we assessed their cytokine production following overnight mitogen stimulation in a subset of four patients. Intratumoural $CD8^+$ and $CD4^+$T cells from FNA were able to produce IFN-γ, TNF-α and IL-2 as well as degranulate (marked by CD107a) (Fig. 3G,H). The tissue-resident fraction of

both $CD8^+$ and $CD4^+$T cells from HCC FNA had enhanced capacity to produce the antitumour cytokines IFN-γ, TNF-α and IL-2 but reduced degranulation potential compared to their non-resident counterparts (Fig. 3I,J). Thus, FNA can be used for functional assessments of TILs, showing that T_{RM} within HCC retain the prototypic enhanced protective effector capacity of T_{RM} in other tissue sites, consistent with their utlity as an immunotherapy target.[36]

We next asked whether the variation in frequencies of T_{RM} within FNA could be atributable to extrinsic regulation within the HCC tumour microenvironment, focusing on $CD11b^+CD33^+HLA\text{-}DR^-CD14^-CD16^+$ neutrophils (PMNs or TANs) that we had noted to be strikingly expanded locally (whereas Tregs were present at a much lower frequency and not enriched locally). We found that the frequency of neutrophils with this suppressive phenotype within the myeloid compartment was robustly inversely correlated with both $CD8^+$ and $CD4^+T_{RM}$ (p = 0.001, Fig. 3K,L).

Notably, these neutrophils were also significantly expanded at baseline among non-responders (progressive disease) compared to patients with subsequent radiological partial or complete response following 3 months of treatment with an anti-PD-L1-based regimen (mean 50.2% *vs.* 25.5%, p = 0.015, Fig. 3M).

In summary, FNA had the capacity to retrieve functional tissue-resident populations compartmentalised within the tumour niche that were inversely correlated with locally sequestered neutrophils expanded in those lacking a treatment response.

FNA reveals local hierarchies of immune checkpoints enriched on T_{RM} in the tumour niche

We postulated that selection of immunotherapies for optimal boosting of antitumour immunity may require analysis of checkpoint targets on T_{RM} rather than circulating T cells. The capacity of FNA to sample the local intensity and hierarchy of expression of different T-cell co-inhibitory receptors was therefore evaluated to assess if it could aid future personalised selection of ICI therapy. We compared global circulating and FNA $CD8^+$T cells for differences in expression of surface inhibitory checkpoint molecules PD-1, TIM-3, LAG-3, TIGIT and 2B4, as well as the metabolic checkpoint CD39 that, together with CD73, generates immunosuppressive adenosine from ATP. Non-linear dimensionality reduction by t-SNE revealed two $CD8^+$T-cell clusters in FNA that were not present in blood (Fig. 4A). The cluster expressing CD69 had intermediate levels of PD-1 with 2B4, whereas the T_{RM} cluster co-expressing both prototypic T_{RM} markers CD69 with CD103 had high expression of PD-1, TIM-3, 2B4 and CD39 (Fig .4A).

We verified this enrichment of checkpoint molecule expression by tissue-resident fractions within $CD8^+$T cells using manual gating of individual inhibitory markers (Fig. 4B and S4A). The current HCC immunotherapy target PD-1 was markedly enriched on tumour $CD8^+T_{RM}$ compared to circulating counterparts, both in percentage and intensity of expression (Fig. 4B). The infiltrating/recirculating $CD69^-CD103^-CD8^+$T cells within tumours and the single-positive $CD69^+CD103^-CD8^+$T cells showed intermediate proportions and intensity of PD-1 expression (Fig. 4B). An analogous stepwise increase in the proportion of $CD8^+$T cells expressing TIM-3, LAG-3, 2B4 and CD39 was seen from blood and tumour-infiltrating to single and double-positive tissue-resident fractions (Fig. S4A). Striking differences were maintained when adjusting for the increased naïve population of T cells in blood by comparing checkpoint molecule expression on just the circulating effector memory subset with intratumoral T_{RM} or effector memory subsets (Fig. 4C and S4B). For example, patients had TIM-3 detectable on less than 1% of their circulating $CD8^+$ effector memory T cells (T_{EM}), underestimating the relevance of this targetable checkpoint within the tumour, where it was expressed on up to 8% of $CD8^+T_{EM}$ and 16% of $CD8^+T_{RM}$ (Fig. 4C and S4B). TIGIT was the exception to this pattern, showing a selective reduction on intratumoral T_{RM} (Fig. 4C), further underscoring their niche-specific co-inhibitory properties. Of note, $CD8^+T_{RM}$ had similar percentages expressing each checkpoint molecule irrespective of underlying disease in the limited number of cases assessed with a single attributable aetiology (viral n = 6, ALD n = 5 and MASLD n = 7, Fig. S4C).

Importantly, there was a lack of concordance between the proportion of circulating $CD8^+$T cells expressing PD-1 and their $CD69^+CD103^+$ tissue-resident counterparts found in tumour FNA (Fig. 4D). The checkpoints TIM-3, LAG-3, 2B4 and CD39 also showed complete discordance in expression between circulating and HCC tissue-resident $CD8^+$T cells (Fig. S4D). Taken together, the profile of checkpoints on blood $CD8^+$T cells was poorly representative of that in the tumour FNA and particularly the $CD8^+T_{RM}$ within it.

$CD4^+$T cells are also increasingly recognised to play critical roles in antitumour immunity,[45,46] so we next examined whether the tissue-resident $CD4^+$T cells identified in FNA also constituted enriched targets for immune checkpoint inhibition. Within the CD4 compartment, there was significant enrichment of PD-1, TIM-3, LAG-3 and CD39 (but not TIGIT) on the intratumoral $CD4^+T_{RM}$ (and PD-1 and TIM-3 on intratumoral $CD4^+T_{EM}$) compared to their circulating $CD4^+$ T_{EM} counterparts (Fig. 4E and S4E).

Combination immunotherapies may be required to rescue T cells co-expressing multiple layers of non-redundant co-inhibitory receptors.[8] We therefore examined the proportion of T cells expressing one, two, three or four of the inhibitory checkpoints currently being targeted in HCC trials (PD-1, TIM-3, LAG-3 and TIGIT). Most peripheral blood $CD8^+$T cells expressed one or less co-inhibitory receptors (predominantly TIGIT>PD-1) with barely any expressing more than two (Fig. 4F). By contrast, $CD8^+T_{RM}$ more commonly co-expressed multiple receptors, with 21.6% expressing more than one and 7.1% $CD8^+T_{RM}$ co-expressing more than two of the four examined co-inhibitory receptors, compared with 7.4% and 0.2% on circulating $CD8^+$T cells (p = 0.0003 and p = 0.0004, respectively, Fig. 4F). The co-expression of multiple ICI targets was similarly enriched on tumour-compartmentalised $CD4^+T_{RM}$ with 16.4% and 4.8% expressing greater than one or two checkpoints, compared to 5.6% and 0.1% in circulating $CD4^+$T cells (p = 0.011 and p = 0.001, Fig. S4F). Taken together, these data underscore the capacity of FNA to reveal locally enriched co-expression of tractable immune checkpoints on T_{RM} compartmentalised within tumours.

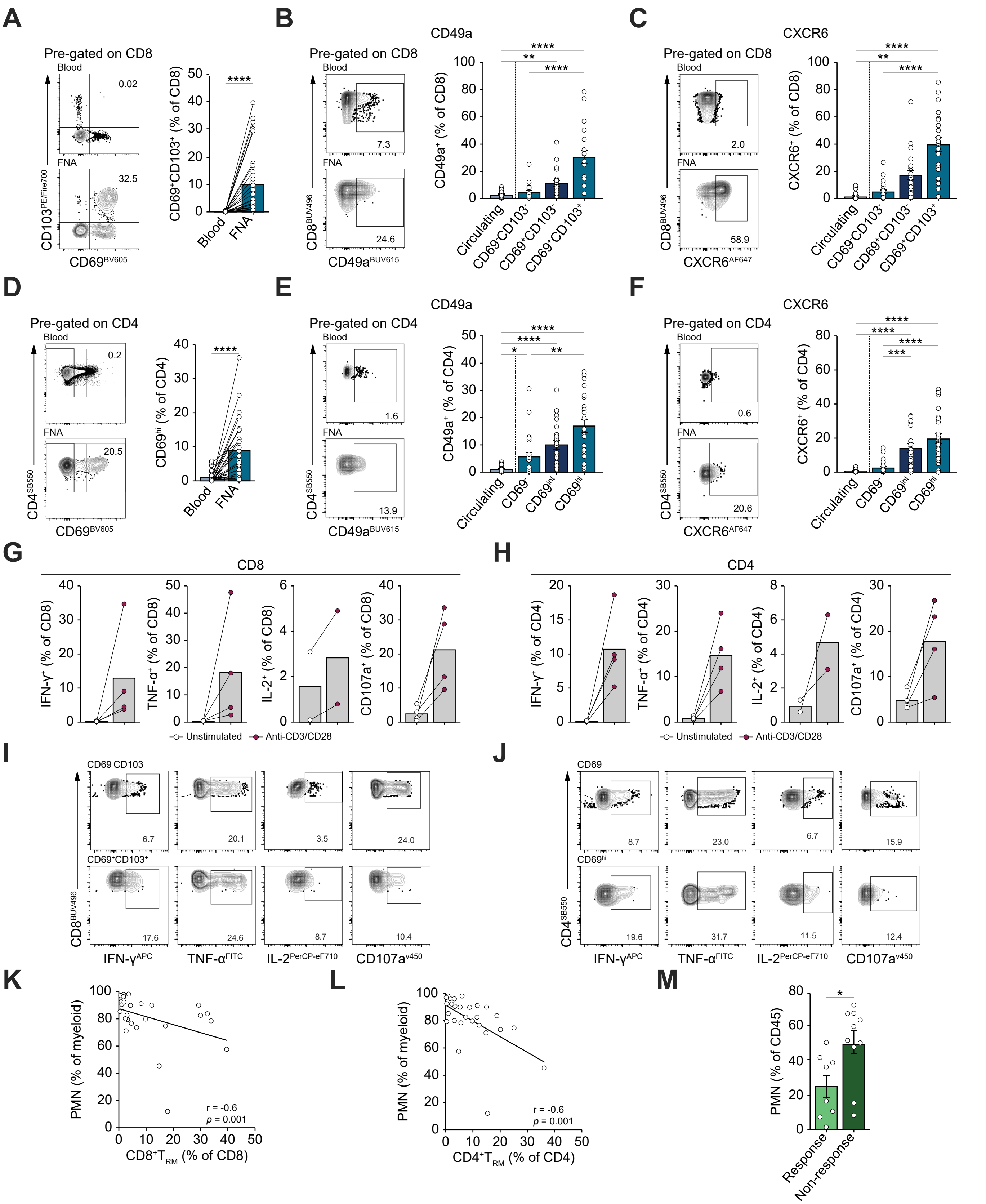

Fig. 3. FNA enables retrieval of tissue-compartmentalised immunity from HCC tumours. (A) Representative flow cytometric plots and summary data comparing the frequency of CD8$^+$T cells with tissue-resident phenotype (CD69$^+$CD103$^+$) in matched blood and FNA, respectively. (B, C) Representative flow cytometric and summary frequency data of CD49a$^+$ (B) or CXCR6$^+$ (C) CD8$^+$T in blood (circulating) compared with tumour-recirculating (CD69$^-$CD103$^-$), single-positive CD69$^+$CD103$^-$ and tissue-resident (CD69$^+$CD103$^+$) CD8$^+$T cells. (D) Representative flow cytometric and summary data comparing frequency of CD4$^+$T cells with CD69^{hi} tissue

In addition to pre-treatment sampling, we postulated that the minimally invasive approach of FNA could be used for repeat sampling to assess the impact of immunotherapy and dissect mechanisms of secondary resistance. In order to interpret any temporal changes in FNA composition, we first assessed the consistency of two samples taken at the same time point. We observed comparable frequencies of all major myeloid and lymphoid immune subsets in the two separate passes taken from the same tumour (Fig. 4G). This congruence suggested the FNA fanning technique applied had overcome any intralesional heterogeneity in HCC to provide consistent sampling. As a proof of principle of the capacity of FNA to detect changes in HCC immune composition following immunotherapy, we then re-sampled one patient after PD-L1/VEGF blockade (atezolizumab/bevacizumab) and, as a control, another who only received a TKI (lenvatinib). In the patient undergoing FNA before and during immunotherapy, there was a contraction in the dominant regulatory population of neutrophils, accompanied by an increase in effector cells (NK cells and $CD3^{+}$T cells), and of particular note, an increase of $CD8^{+}T_{RM}$ 12 weeks after starting immunotherapy (Fig. 4H). By contrast, the patient receiving lenvatinib showed an expansion in neutrophils and no expansion of antitumour effectors, including $CD8^{+}T_{RM}$, when comparing their baseline and on treatment FNA (Fig. 4H). These examples demonstrate the capacity of serial FNA to sample consistent immune profiles when taken contemporaneously and to monitor changes in immune profiles when taken before and after the introduction of immunotherapy.

Discussion

Immunotherapy is transforming the treatment potential of many tumours, including HCC. However, there is a pressing need for assays to guide biomarker discovery and selection of those most likely to benefit from approved and emerging immunotherapy combinations so as to avoid unnecessary toxicity in non-responders. With the advent of additional ICIs, such as CTLA-4 inhibitors for HCC, and others being evaluated in clinical trials, targeted selection has become more pressing. Selection based on tumour expression of PD-L1 is not a reliable biomarker for responsiveness to PD-1 blockade in HCC[8,47] but the utility of selection based on the hierarchical expression of PD-1 or other checkpoint receptors on T cells compartmentalised within the tumour has not been tested. Even with optimal selection of combination ICI, alternative immunotherapeutic approaches will still be necessary;[48] their identification requires comprehensive immune profiling of the tumour niche before and after ICI therapy. Here we have shown that FNA provides a minimally invasive approach to tackling these unmet needs in HCC immunotherapy.

Accumulating data have highlighted a critical role for $CD8^{+}T_{RM}$ in the control of many tumours, including HCC,[10,17,18,37] implying that ICI selection should take account of their ability to rescue these tumour-compartmentalised populations. Our finding of ICI receptors enriched on the tissue-resident component of TILs underscores the advantage of sampling the immune profile at the tumour site rather than just the periphery. Using FNA, we demonstrated that most checkpoints examined were markedly enriched on $CD8^{+}T_{RM}$ within the HCC niche. Importantly, there was major discordance between their expression on peripheral effector memory and tissue-compartmentalised $CD8^{+}$T cells, indicating that levels on blood T cells cannot be used as a surrogate for their relative dominance in the tumour and may therefore fail to predict the suitability of a particular ICI to target the critical component compartmentalised in HCC. Similarly, there was a lack of congruence between the blood and tumour-resident compartment in the proportion of $CD8^{+}$T cells expressing multiple co-inhibitory receptors, suggesting FNA would provide more accurate information for the optimal application of combination regimens. Although ICI design has been primarily focused on the rescue of $CD8^{+}$T cells, our data also reveal that many $CD4^{+}$T cells resident within HCC express PD-1 and other checkpoints – at different levels and with more co-expression than their effector memory counterparts in the periphery – supporting simultaneous evaluation of these important antitumour effectors as therapeutic targets.

The multiparametric power of spectral flow cytometry allowed multiple innate and adaptive effectors and regulators to be quantified and phenotyped by *ex vivo* staining of a single well of cells. For example, we identified intratumoral DCs and Tfh cells, whose role within HCC merits further study; a recent study has highlighted a role for $CXCL10^{+}$ macrophages and $PD\text{-}1^{-}CD45RA^{+}T_{EM}$ that could be incorporated in future panels. We found neutrophils, with an immunosuppressive phenotype previously ascribed to gMDSC,[34,35,49] to be the dominant population within many HCC FNA. Recent studies have described a suppressive role for neutrophils in HCC,[12,35,50–53] suggesting that their highly variable frequency within tumour FNA (which was not predictable from the blood) may associate with prognosis and/or response to immunotherapy. Consistent with this, we observed a significant association with their intratumoral expansion and non-response to immunotherapy in our cohort. Importantly, we also found that frequencies of these neutrophils had a strong negative correlation with both $CD4^{+}$ and $CD8^{+}T_{RM}$, providing mechanistic insights into their association with treatment outcome. Their reduction, coinciding with expansion in $CD8^{+}T_{RM}$, in a patient given PD-L1/VEGF blockade (and not in one given a TKI alone) raises the possibility they may be partially targeted;

residency phenotype in blood and FNA. (E,F) Representative flow cytometric and summary data showing the frequency of $CD49a^{+}$ (E) or $CXCR6^{+}$ (F) cells within $CD4^{+}$T cells circulating in blood, infiltrating ($CD69^{-}$), CD69-intermediate ($CD69^{int}$) and tissue-resident ($CD69^{hi}$) $CD4^{+}$T cells. (G,H) Frequency of global $CD8^{+}$ (G) and $CD4^{+}$ (H) T cells within FNA producing effector molecules IFN-γ (n = 4), TNF-α (n = 4), IL-2 (n = 2) and CD107a (n = 4) after 16 h stimulation with anti-CD3/CD28. (I, J) Example flow cytometric plots from one donor comparing production of IFN-γ, TNF-α, IL-2 and CD107a between tumour-recirculating and tissue-resident subsets of $CD8^{+}$ (I) and $CD4^{+}$ (J) T cells. (K, L) Correlation between $CD16^{+}$ PMN and $CD8^{+}$ (K) and $CD4^{+}$ (L) T_{RM}. (M) Baseline frequency of $CD16^{+}$ PMN between donors grouped by treatment outcome after 12 weeks of anti-PD-L1; response (complete or partial response [n = 8]) *vs*. non-response (progressive disease [n = 10]). Data shown are mean ± SEM; significance assessed by non-parametric *t* test (A,D,M); one-way ANOVA with Dunn's *post hoc* test for multiple comparisons (B, C, E, F). (G, H) No statistical analysis was performed due to limited sample size. *p <0.05, **p <0.01, ***p <0.001, ****p <0.0001 or Spearman correlation (K, L). FNA, fine needle aspiration; HCC, hepatocellular carcinoma; MNP, mononuclear phagocyte; PMN, polymorphonuclear cell; T_{RM}, tissue-resident memory T cell.

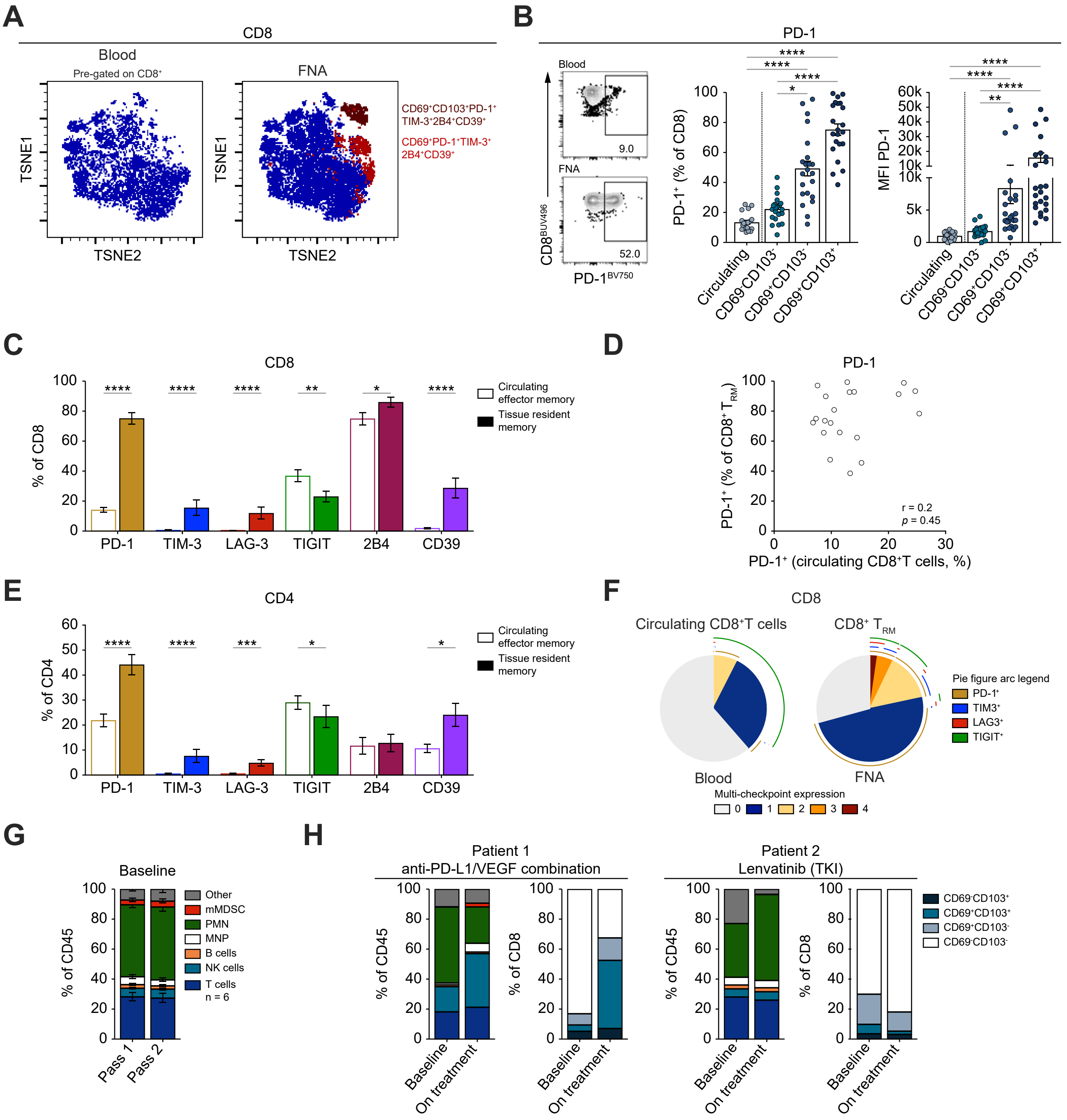

Fig. 4. Enrichment of inhibitory pathways in HCC. (A) t-SNE representation of global CD8+T cells in blood and matched FNA clustered according to flow cytometric staining of residency and exhaustion markers in a sample of four patients. (B) Representative flow cytometric plots of PD-1+CD8+T cells in blood and FNA with summary data showing percentage and expression by MFI by circulating *vs.* tumour-recirculating (CD69-CD103-), single-positive CD69+CD103-, or tissue-resident (CD69+CD103+) CD8+T cells in FNA. (C) Percentage of effector memory (CD62L-) CD8+T$_{EM}$ cells in blood and FNA CD8+T$_{RM}$ expressing PD-1, TIM-3, LAG-3, TIGIT, 2B4 or CD39 (n = 22 assessed with this panel). (D) Scatter plot showing relationship between the frequency of PD-1+ subsets within circulating CD8+T cells and CD8+T$_{RM}$ in FNA. (E) Frequency of circulating CD4+T$_{EM}$ *vs.* FNA CD4+T$_{RM}$ expressing PD-1, TIM-3, LAG-3, TIGIT, 2B4 or CD39 (n = 22). (F) SPICE chart representation of the proportions and combinations of inhibitory checkpoints expressed by CD8+T cells in blood (global) *vs.* FNA (T$_{RM}$). Coloured slices indicate the number of checkpoints expressed; colour of arc indicates identity of checkpoint molecule expressed (n = 16). (G) Summary data assessing frequency of immune subsets within two separately analysed FNA passes acquired at the same time point (n = 6). (H) Summary data comparing immune subsets identified by FNA in two example patients sampled at baseline, and whilst on therapy with either combination anti-PDL1/VEGF (n = 1) or the TKI lenvatinib (n = 1). Immune subset frequencies and proportions of infiltrating, single-positive CD69+CD103- or CD69-CD103+ and double-positive CD69+CD103+ within the CD8 compartment shown. Data shown are mean ± SEM with significance assessed by one-way ANOVA with Dunn's *post hoc* test (B), multiple *t* tests and non-parametric tests (C, E) or non-parametric Spearman correlation (D). **p* <0.05, ***p* <0.01, ****p* <0.001, *****p* <0.0001. FNA, fine needle aspiration; HCC, hepatocellular carcinoma; MFI, mean fluorescence intensity; SPICE, simplified presentation of incredibly complex evaluations; T$_{EM}$, effector memory T; TKI, tyrosine kinase inhibitor; T$_{RM}$, tissue-resident memory T cell.

further phenotypic and functional studies are required to assess their suppressive capacity and identify specific therapeutic targets.

An obvious limitation of FNA is that they do not allow histological examination of the tumour and its margin or the topology of immune infiltrates and their cellular interactions, as provided by sections from core biopsies. FNA have previously been used for cytological diagnosis of HCC,[28,29] but it is likely that biopsies will largely remain the mainstay for initial HCC grading, allowing additional spatial immune characterisation. However, we found that FNA provided a higher yield of leukocytes than biopsies, allowing more reliable and comprehensive immunophenotyping, as reported previously in mesothelioma.[54] Biopsies and FNA are both liable to sampling error, but the fanning technique allows FNA to aspirate a wider tumour field, whilst avoiding immune contamination from the surrounding unaffected tissue that we and others find is often included in conventional core biopsies.[14,21,30,31] Because of its minimally invasive nature, FNA could also be used to compare the surrounding liver and intratumoral immune niche and to sample multifocal HCC. Both FNA and biopsy carry a minimal theoretical risk of tumour seeding, so we restricted their use to the advanced HCC setting.[19,28]

Two separate but contemporaneous passes showed consistent immune profiles, validating the interpretation of changes seen on longitudinal FNA. Serial FNA sampling of the liver is well tolerated due to its minimally invasive nature and has been successfully applied to monitor therapeutic responses in viral hepatitis, including in several HBV functional cure trials.[21,26,27,55] Thus, we propose that FNA of liver tumours could be incorporated as an adjunct to baseline biopsy to assess tumour immune landscapes both before and during immunotherapy. Larger cohorts will be needed to examine whether tumour immune profiles obtained by FNA provide reliable biomarkers for the selection of patients for different ICI regimens and to extend the examination of any influences of underlying aetiology on neutrophils and other mediators of resistance. Unlike biopsies, FNA samples do not require tissue processing and provide larger yields of leukocytes; immediate flow cytometric analysis removes the need for specialist laboratories and provides results within a few hours. Up to 4 FNA passes have been taken in other settings,[22,27] allowing more detailed phenotypic dissection of relevant cell types, including by single-cell RNA sequencing, as well as functional assays.[25–27,56] Here we exemplify the utility of FNA for functional assays, eliciting the first insights into the distinct functional features of T_{RM} within HCC. We show that sufficient functional T cells can be isolated by FNA for future *in vitro* or *ex vivo* assessment of effector responses to different immunotherapy strategies for optimal treatment personalisation. Our findings are also applicable to other solid cancers, many of which show strong associations with $CD8^{+}T_{RM}$ frequencies; however, the few tumour FNA immunophenotyping studies published to date[54,57] have not attempted to identify these cells.

In summary, we show that FNA can comprehensively profile the local immune landscape of advanced HCC, providing a minimally invasive and rapid assay. Our data confirm that FNA can sample tumour-compartmentalised $CD4^{+}$ and $CD8^{+}T_{RM}$ that we find are markedly enriched for multiple checkpoints, with discordant expression from their circulating counterparts. The rapid, simple processing of FNA also enabled detection of locally enriched but highly variable proportions of fragile neutrophils, that we show associate with non-responsiveness to ICI and correlate inversely with $CD4^{+}$ and $CD8^{+}T_{RM}$; their response to current trials with CXCR2 inhibitors could be assessed by serial FNA.[50,58] Thus, we conclude that FNA provide a valuable tool to more fully define the heterogeneity of local immunity in liver tumours of different aetiologies and the dynamic changes resulting from treatment. We suggest FNA could be tested in larger cohorts and clinical trial settings to personalise the selection of patients for existing and novel immunotherapy combinations and/or identify tractable primary and secondary resistance mechanisms based on tumour-compartmentalised immunity.

Affiliations

[1]*Institute of Immunity and Transplantation, UCL, London, United Kingdom;* [2]*Department of Radiology, Royal Free London, United Kingdom;* [3]*Centre for Immunobiology, Blizard Institute, Queen Mary University of London, United Kingdom;* [4]*Cancer Institute, UCL, London, United Kingdom*

Abbreviations

ALD, alcohol-associated liver disease; DC, dendritic cell; FNA, fine needle aspiration; gMDSC, granulocytic myeloid-derived suppressor cell; HCC, hepatocellular carcinoma; ICI, immune checkpoint inhibitor; mAbs, monoclonal antibodies; MASLD, metabolic dysfunction-associated steatotic liver disease; mMDSC, monocytic myeloid-derived suppressor cell; MNP, mononuclear phagocyte; NK cell, natural killer cell; PBMC, peripheral blood mononuclear cell; PMN, polymorphonuclear neutrophil; TAN, tumour-associated neutrophil; T_{EM}, effector memory T cell; Tfh, T follicular helper; TIL, tumour-infiltrating lymphocyte; TKI, tyrosine kinase inhibitor; Treg, regulator T cell; T_{RM}, tissue-resident memory T cell.

Financial support

This study was funded by the CRUK Hunter Accelerator Award to GA-M, TM, MKM and Wellcome Trust Investigator award and Royal Free Charity grant to MKM, MD and TM.

Conflicts of interest

The Maini lab has received unrestricted funding from Gilead Sciences and MKM has joined hepatitis B advisory boards for Gilead, Astrivax, GSK, Moderna, Roche. TM has contributed to advisory board for Roche, Astra Zeneca, Guerbet, Ipsen, GreyWolf, Signant Health, Parabilis Medicines and has Insitution research grants from MSD, Bayer and Boston Scientific. UG has joined an advisory board for GSK.

Please refer to the accompanying ICMJE disclosure forms for further details.

Authors' contributions

TM, MKM conceived the study and obtained funding, GA-M, NS, LJP, MD, MKM designed experiments, GA-M, SK, TM-S, YZ, DBR, JD carried out experiments, GA-M, LS analysed data, VN, RS, SK, GA-M, AC, UG, EG, TM provided clinical samples and expertise, GA-M, TM, MKM wrote the manuscript, all other authors provided critical review of the manuscript.

Data availability

Data will be made available upon request.

Acknowledgements

The authors are grateful to all patients who participated and clinical staff of the Joint Hepatology HCC service at The Royal Free London for their help with recruitment. We are grateful for funding from CRUK, Wellcome and Royal Free Chariy. We also acknowledge the support staff at the Institute of Immunity and Transplantation Flow Cytometry Core Facility, UCL.

Supplementary data

Supplementary data to this article can be found online at https://doi.org/10.1016/j.jhepr.2025.101637.

References

Author names in bold designate shared co-first authorship

[1] Rumgay H, Ferlay J, de Martel C, et al. Global, regional and national burden of primary liver cancer by subtype. Eur J Cancer 2022;161:108–118. https://doi.org/10.1016/j.ejca.2021.11.023.
[2] Llovet JM, Kelley RK, Villanueva A, et al. Hepatocellular carcinoma. Nat Rev Dis Primers 2021;7:6. https://doi.org/10.1038/s41572-020-00240-3.
[3] Cheng A-L, Qin S, Ikeda M, et al. Updated efficacy and safety data from IMbrave150: atezolizumab plus bevacizumab vs. sorafenib for unresectable hepatocellular carcinoma. J Hepatol 2022;76:862–873. https://doi.org/10.1016/j.jhep.2021.11.030.
[4] Finn RS, Qin S, Ikeda M, et al. Atezolizumab plus bevacizumab in unresectable hepatocellular carcinoma. N Engl J Med 2020;382:1894–1905. https://doi.org/10.1056/NEJMoa1915745.
[5] Abou-Alfa GK, Lau G, Kudo M, et al. Tremelimumab plus durvalumab in unresectable hepatocellular carcinoma. NEJM Evid 2022;1. https://doi.org/10.1056/EVIDoa2100070.
[6] Sangro B, Chan SL, Kelley RK, et al. Four-year overall survival update from the phase III HIMALAYA study of tremelimumab plus durvalumab in unresectable hepatocellular carcinoma. Ann Oncol 2024;35:448–457. https://doi.org/10.1016/j.annonc.2024.02.005.
[7] Rimassa L, Finn RS, Sangro B. Combination immunotherapy for hepatocellular carcinoma. J Hepatol 2023;79:506–515. https://doi.org/10.1016/j.jhep.2023.03.003.
[8] Childs A, Aidoo-Micah G, Maini MK, et al. Immunotherapy for hepatocellular carcinoma. JHEP Rep 2024;6:101130. https://doi.org/10.1016/j.jhepr.2024.101130.
[9] Llovet JM, Castet F, Heikenwalder M, et al. Immunotherapies for hepatocellular carcinoma. Nat Rev Clin Oncol 2022;19:151–172. https://doi.org/10.1038/s41571-021-00573-2.
[10] Lim CJ, Lee YH, Pan L, et al. Multidimensional analyses reveal distinct immune microenvironment in hepatitis B virus-related hepatocellular carcinoma. Gut 2019;68:916–927. https://doi.org/10.1136/gutjnl-2018-316510.
[11] Cappuyns S, Philips G, Vandecaveye V, et al. PD-1- CD45RA+ effector-memory CD8 T cells and CXCL10+ macrophages are associated with response to atezolizumab plus bevacizumab in advanced hepatocellular carcinoma. Nat Commun 2023;14:7825. https://doi.org/10.1038/s41467-023-43381-1.
[12] Liu S, Zhou Y, Li G, et al. PLAUR+ neutrophils drive anti-PD-1 therapy resistance in patients with hepatocellular carcinoma by Shaping an immunosuppressive microenvironment. Adv Sci 2025. https://doi.org/10.1002/advs.202507167.
[13] **Park M-S**, **Jo H**, **Kim H**, et al. Molecular landscape of tumor-associated tissue-resident memory T cells in tumor microenvironment of hepatocellular carcinoma. Cell Commun Signaling 2025;23:80. https://doi.org/10.1186/s12964-025-02070-w.
[14] **Blaise L**, **Ziol M**, Campani C, et al. Utility of tumor and non-tumor biopsies during percutaneous radiofrequency ablation for hepatocellular carcinoma. JHEP Rep 2025:101430. https://doi.org/10.1016/j.jhepr.2025.101430.
[15] Huang AC, Postow MA, Orlowski RJ, et al. T-cell invigoration to tumour burden ratio associated with anti-PD-1 response. Nature 2017;545:60–65. https://doi.org/10.1038/nature22079.
[16] Pallett LJ, Maini MK. Liver-resident memory T cells: life in lockdown. Semin Immunopathol 2022;44:813–825. https://doi.org/10.1007/s00281-022-00932-w.
[17] Barsch M, Salié H, Schlaak AE, et al. T-cell exhaustion and residency dynamics inform clinical outcomes in hepatocellular carcinoma. J Hepatol 2022;77:397–409. https://doi.org/10.1016/j.jhep.2022.02.032.
[18] Okła K, Farber DL, Zou W. Tissue-resident memory T cells in tumor immunity and immunotherapy. J Exp Med 2021;218. https://doi.org/10.1084/jem.20201605.
[19] Sangro B, Argemi J, Ronot M, et al. EASL Clinical Practice Guidelines on the management of hepatocellular carcinoma. J Hepatol 2025;82:315–374. https://doi.org/10.1016/j.jhep.2024.08.028.
[20] Childs A, Zakeri N, Ma YT, et al. Biopsy for advanced hepatocellular carcinoma: results of a multicentre UK audit. Br J Cancer 2021;125:1350–1355. https://doi.org/10.1038/s41416-021-01535-2.
[21] Gill US, Pallett LJ, Thomas N, et al. Fine needle aspirates comprehensively sample intrahepatic immunity. Gut 2019;68:1493–1503. https://doi.org/10.1136/gutjnl-2018-317071.
[22] Pembroke T, Gallimore A, Godkin A. Tracking the kinetics of intrahepatic immune responses by repeated fine needle aspiration of the liver. J Immunol Methods 2015;424:131–135. https://doi.org/10.1016/j.jim.2015.04.011.
[23] Spaan M, van Oord GW, Janssen HLA, et al. Longitudinal analysis of peripheral and intrahepatic NK cells in chronic HCV patients during antiviral therapy. Antivir Res 2015;123:86–92. https://doi.org/10.1016/j.antiviral.2015.09.006.
[24] Sprengers D, van der Molen RG, Kusters JG, et al. Flow cytometry of fine-needle-aspiration biopsies: a new method to monitor the intrahepatic immunological environment in chronic viral hepatitis. J Viral Hepat 2005;12:507–512. https://doi.org/10.1111/j.1365-2893.2005.00626.x.
[25] Kim SC, Wallin JJ, Ghosheh Y, et al. Efficacy of antiviral therapy and host–virus interactions visualised using serial liver sampling with fine-needle aspirates. JHEP Rep 2023;5:100817. https://doi.org/10.1016/j.jhepr.2023.100817.
[26] Nkongolo S, Mahamed D, Kuipery A, et al. Longitudinal liver sampling in patients with chronic hepatitis B starting antiviral therapy reveals hepatotoxic CD8+ T cells. J Clin Invest 2023;133. https://doi.org/10.1172/JCI158903.
[27] Genshaft AS, Subudhi S, Keo A, et al. Single-cell RNA sequencing of liver fine-needle aspirates captures immune diversity in the blood and liver in chronic hepatitis B patients. Hepatology 2023;78:1525–1541. https://doi.org/10.1097/HEP.0000000000000438.
[28] Wee A. Fine-needle aspiration biopsy of hepatocellular carcinoma and related hepatocellular nodular lesions in cirrhosis: controversies, challenges, and expectations. Patholog Res Int 2011;2011:1–17. https://doi.org/10.4061/2011/587936.
[29] Caturelli E, Solmi L, Anti M, et al. Ultrasound guided fine needle biopsy of early hepatocellular carcinoma complicating liver cirrhosis: a multicentre study. Gut 2004;53:1356–1362. https://doi.org/10.1136/gut.2003.032359.
[30] Lee JM, Lee HS, Hyun JJ, et al. Slow-pull using a fanning technique is more useful than the standard suction technique in EUS-guided fine needle aspiration in pancreatic masses. Gut Liver 2018;12:360–366. https://doi.org/10.5009/gnl17140.
[31] Bang J, Magee S, Ramesh J, et al. Randomized trial comparing fanning with standard technique for endoscopic ultrasound-guided fine-needle aspiration of solid pancreatic mass lesions. Endoscopy 2013;45:445–450. https://doi.org/10.1055/s-0032-1326268.
[32] **Cossarizza A**, **Chang H**, **Radbruch A**, et al. Guidelines for the use of flow cytometry and cell sorting in immunological studies (third edition). Eur J Immunol 2021;51:2708–3145. https://doi.org/10.1002/eji.202170126.
[33] Guilliams M, Mildner A, Yona S. Developmental and functional heterogeneity of monocytes. Immunity 2018;49:595–613. https://doi.org/10.1016/j.immuni.2018.10.005.
[34] Pallett LJ, Gill US, Quaglia A, et al. Metabolic regulation of hepatitis B immunopathology by myeloid-derived suppressor cells. Nat Med 2015;21:591–600. https://doi.org/10.1038/nm.3856.
[35] Veglia F, Sanseviero E, Gabrilovich DI. Myeloid-derived suppressor cells in the era of increasing myeloid cell diversity. Nat Rev Immunol 2021;21:485–498. https://doi.org/10.1038/s41577-020-00490-y.
[36] Zakeri N, Hall A, Swadling L, et al. Characterisation and induction of tissue-resident gamma delta T-cells to target hepatocellular carcinoma. Nat Commun 2022;13:1372. https://doi.org/10.1038/s41467-022-29012-1.
[37] Barsch M, Salié H, Mesesan A, et al. T cells in the heterogeneous tumour immune microenvironment of hepatocellular carcinoma: implications for immune checkpoint inhibitor therapy. Liver Cancer Int 2023;4:58–72. https://doi.org/10.1002/lci2.72.
[38] Pallett LJ, Davies J, Colbeck EJ, et al. IL-2high tissue-resident T cells in the human liver: sentinels for hepatotropic infection. J Exp Med 2017;214:1567–1580. https://doi.org/10.1084/jem.20162115.
[39] Richter MV, Topham DJ. The alpha1beta1 integrin and TNF receptor II protect airway CD8+ effector T cells from apoptosis during influenza infection. J Immunol 2007;179:5054–5063. https://doi.org/10.4049/jimmunol.179.8.5054.
[40] Cheuk S, Schlums H, Gallais Sérézal I, et al. CD49a expression defines tissue-resident CD8 + T cells poised for cytotoxic function in human skin. Immunity 2017;46:287–300. https://doi.org/10.1016/j.immuni.2017.01.009.
[41] **Kim JH**, **Han JW**, Choi YJ, et al. Functions of human liver CD69+CD103-CD8+ T cells depend on HIF-2α activity in healthy and pathologic livers. J Hepatol 2020;72:1170–1181. https://doi.org/10.1016/j.jhep.2020.01.010.
[42] Heydtmann M, Lalor PF, Eksteen JA, et al. CXC chemokine Ligand 16 promotes integrin-mediated adhesion of liver-infiltrating lymphocytes to

cholangiocytes and hepatocytes within the inflamed human liver. J Immunol 2005;174:1055–1062. https://doi.org/10.4049/jimmunol.174.2.1055.
[43] Mossanen JC, Kohlhepp M, Wehr A, et al. CXCR6 inhibits hepatocarcinogenesis by promoting natural killer T- and CD4+ T-cell–dependent control of senescence. Gastroenterology 2019;156:1877–1889.e4. https://doi.org/10.1053/j.gastro.2019.01.247.
[44] Wiggins BG, Pallett LJ, Li X, et al. The human liver microenvironment shapes the homing and function of CD4 $^{+}$ T-cell populations. Gut 2022;71:1399–1411. https://doi.org/10.1136/gutjnl-2020-323771.
[45] **Cachot A**, **Bilous M**, **Liu Y-C**, et al. Tumor-specific cytolytic CD4 T cells mediate immunity against human cancer. Sci Adv 2021;7. https://doi.org/10.1126/sciadv.abe3348.
[46] Magen A, Hamon P, Fiaschi N, et al. Intratumoral dendritic cell–CD4+ T helper cell niches enable CD8+ T cell differentiation following PD-1 blockade in hepatocellular carcinoma. Nat Med 2023;29:1389–1399. https://doi.org/10.1038/s41591-023-02345-0.
[47] Zhu AX, Abbas AR, de Galarreta MR, et al. Molecular correlates of clinical response and resistance to atezolizumab in combination with bevacizumab in advanced hepatocellular carcinoma. Nat Med 2022;28:1599–1611. https://doi.org/10.1038/s41591-022-01868-2.
[48] Zhou G, Sprengers D, Boor PPC, et al. Antibodies against immune checkpoint molecules restore functions of tumor-infiltrating T cells in hepatocellular carcinomas. Gastroenterology 2017;153:1107–1119.e10. https://doi.org/10.1053/j.gastro.2017.06.017.
[49] Akkari L, Amit I, Bronte V, et al. Defining myeloid-derived suppressor cells. Nat Rev Immunol 2024;24:850–857. https://doi.org/10.1038/s41577-024-01062-0.
[50] Leslie J, Mackey JBG, Jamieson T, et al. CXCR2 inhibition enables NASH-HCC immunotherapy. Gut 2022;71:2093–2106. https://doi.org/10.1136/gutjnl-2021-326259.
[51] Ma C, Zhang Q, Greten TF. MDSCs in liver cancer: a critical tumor-promoting player and a potential therapeutic target. Cell Immunol 2021;361:104295. https://doi.org/10.1016/j.cellimm.2021.104295.
[52] **Meng Y**, **Ye F**, **Nie P**, et al. Immunosuppressive CD10+ALPL+ neutrophils promote resistance to anti-PD-1 therapy in HCC by mediating irreversible exhaustion of T cells. J Hepatol 2023;79:1435–1449. https://doi.org/10.1016/j.jhep.2023.08.024.
[53] Geh D, Leslie J, Rumney R, et al. Neutrophils as potential therapeutic targets in hepatocellular carcinoma. Nat Rev Gastroenterol Hepatol 2022;19:257–273. https://doi.org/10.1038/s41575-021-00568-5.
[54] Lizotte PH, Jones RE, Keogh L, et al. Fine needle aspirate flow cytometric phenotyping characterizes immunosuppressive nature of the mesothelioma microenvironment. Sci Rep 2016;6:31745. https://doi.org/10.1038/srep31745.
[55] **Testoni B**, **Roca Suarez AA**, Battisti A, et al. Evaluation of the HBV liver reservoir with fine needle aspirates. JHEP Rep 2023;5:100841. https://doi.org/10.1016/j.jhepr.2023.100841.
[56] Roy-Chowdhuri S, Chen H, Singh RR, et al. Concurrent fine needle aspirations and core needle biopsies: a comparative study of substrates for next-generation sequencing in solid organ malignancies. Mod Pathol 2017;30:499–508. https://doi.org/10.1038/modpathol.2016.228.
[57] Franzén B, Viktorsson K, Kamali C, et al. Multiplex immune protein profiling of fine-needle aspirates from patients with non-small-cell lung cancer reveals signatures associated with PD-L1 expression and tumor stage. Mol Oncol 2021;15:2941–2957. https://doi.org/10.1002/1878-0261.12952.
[58] Evans J. Clinical trial of whether AZD5069 combined with immunotherapy (durvalumab) is effective for patients with advanced primary liver cancer. Http://IsrctnCom/n.d. https://doi.org/10.1186/ISRCTN12669009.

Keywords: Hepatocellular carcinoma (HCC); fine needle aspiration; tissue-resident T cells; checkpoint inhibitors; PD-1; tumour immunity; neutrophils (PMN or TAN); gMDSC.

Received 27 February 2025; received in revised form 6 October 2025; accepted 10 October 2025; Available online 16 October 2025

Journal of Hepatology, Volume *8*

Supplemental information

Fine needle aspirates characterise the hepatocellular carcinoma immune niche to predict immune checkpoint inhibitor outcomes

Gloryanne Aidoo-Micah, Stephanie Kucykowicz, Nathalie Schmidt, Vishnu Naidu, Rushabh Shah, Sayani Khara, Tate Mckinnon-Snell, Yiya Zhong, Daniel Brown Romero, Jessica Davies, Laura Pallett, Leo Swadling, Mariana Diniz, Alexa Childs, Upkar Gill, Edward Green, Tim Meyer, and Mala K. Maini

Fine needle aspirates characterise the hepatocellular carcinoma immune niche to predict immune checkpoint inhibitor outcomes

Gloryanne Aidoo-Micah, Stephanie Kucykowicz, Nathalie Schmidt, Vishnu Naidu, Rushabh Shah, Sayani Khara, Tate Mckinnon-Snell, Yiya Zhong, Daniel Brown Romero, Jessica Davies, Laura Pallett, Leo Swadling, Mariana Diniz, Alexa Childs, Upkar Gill, Edward Green, Tim Meyer, Mala Maini

Table of contents

Fig. S1. 2

Fig. S2. 4

Fig. S3 5

Fig. S4 6

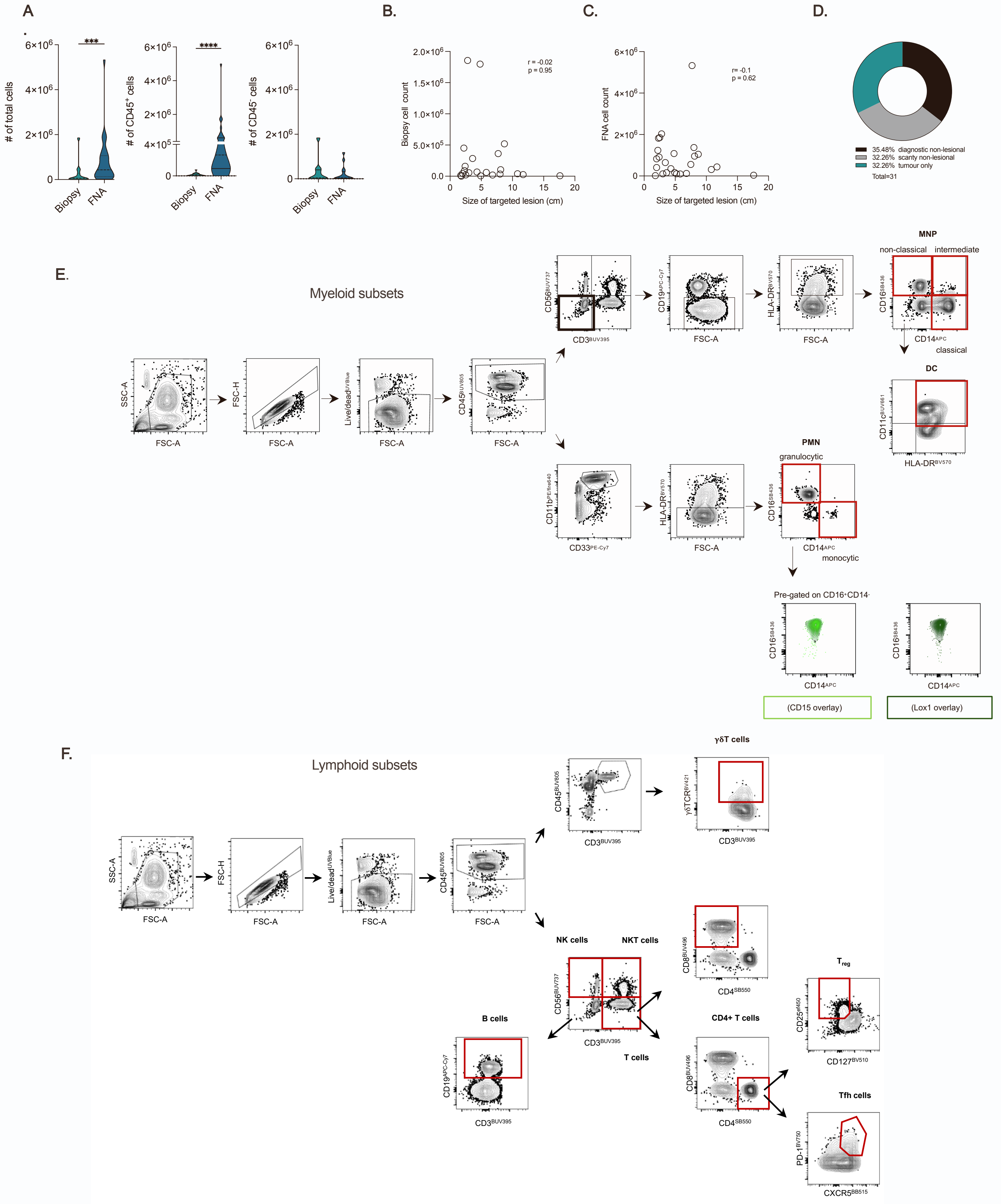

Fig. S1. Identification of immune subsets in HCC. (A) Total number of cells, immune (CD45$^+$) and non-immune (CD45$^-$) obtained from biopsy and FNA, as

quantified by flow cytometric acquisition of whole sample. (b, c) Scatter plots of relationship between the size of HCC and total number of cells from biopsy (B) and FNA (C). (D) Proportion of tumour biopsies containing non-lesional tissue, as reported by routine histological assessment of biopsies from all enrolled patients. (E,F) Representative flow cytometric plots showing sequential manual gating strategy for identification of myeloid (E) and lymphoid (F) subsets. Cells were identified by size (forward scatter area, FSC-A) and granularity (side scatter area; SSC-A), doublet exclusion (forward scatter height; FSC-H), viability staining of live cells and CD45+ immune cells, with subsequent sequential identification of monocytes, myeloid derived suppressor cells (MDSC) and dendritic cells (DC). Overlay of CD15 and Lox1 expression on $CD16^+$ PMN, indicating an overlapping phenotype consistent with suppressive immature PMN (E); and gamma delta (γδ) T cells; $CD56^+CD3^-$ natural killer (NK) cells, $CD56^+CD3^+$ NKT cells, $CD3^+CD56^-$ conventional $CD8^+$T cells, and $CD4^+$T cells, $CD25^+CD127^{lo}$ $CD4^+$ T cells (Treg), $CD19^+$ B cells, and $PD1^+CXCR5^+$T follicular helper (Tfh) cells (F). Significance was determined by non-parametric t-test, Mann-Whitney (A), and nonparametric Spearman correlation (B,C). ***p<0.001,****p<0.0001

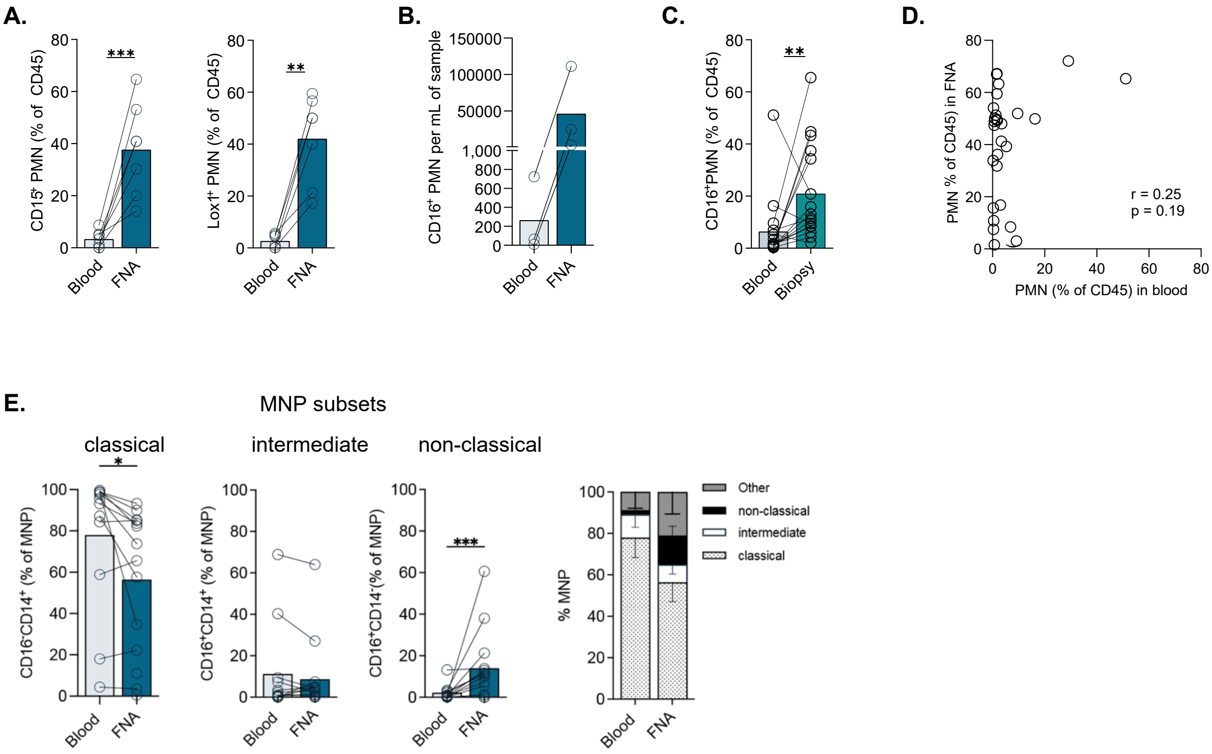

Fig. S2. Differences within the myeloid compartments in blood and tissue. (A) Frequency of immature PMN identified based on expression of CD15 ($CD11b^+CD33^+HLADR^-CD15^+CD14^-$) or Lox1 ($CD11b^+CD33^+HLADR^-Lox1^+CD14^-$) between matched blood and FNA in a subset of samples co-stained for these markers (n=7). (B) Absolute counts of $CD16^+$ PMN within blood and FNA presented as number of cells per mL of sample using counting beads (n=3). (C) Frequency comparison of $CD16^+$ PMN in blood and biopsy. (D) Scatter plot showing correlation between the frequency of $CD16^+$ PMN in blood v FNA. (E) Frequency comparison of classical ($CD16^-CD14^+$), intermediate ($CD16^+CD14^+$) and non-classical ($CD16^+CD14^-$) MNP within matched blood and FNA samples (n=11). Data represent mean with significance determined by non-parametric t-test, Wilcoxin test. *p<0.05, **p<0.01, ***p<0.001,****p<0.0001 (A-C, E) and nonparametric Spearman correlation (D).

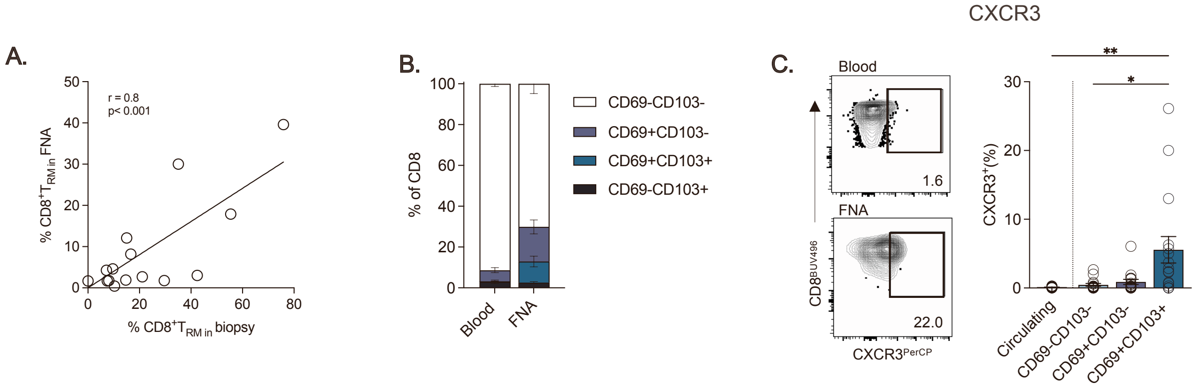

Fig. S3. FNA sampling of tissue compartmentalised immune cells. (A) Scatter plot showing relationship between frequency of $CD8^{+}T_{RM}$ from matched FNA and biopsy(n=15). (B) Summary data of non-resident tumour infiltrating/recirculating ($CD69^{-}CD103^{-}$), single positive $CD69^{+}CD103^{-}$, $CD69^{-}CD103^{+}$ and tissue-resident $CD69^{+}CD103^{+}$ subsets among $CD8^{+}$T cells (n=22). (C) Representative flow cytometric and summary data showing the frequency of $CXCR3^{+}$ cells within $CD8^{+}$T cells circulating in blood, infiltrating ($CD69^{-}CD103^{-}$), $CD69^{+}CD103^{-}$ and tissue-resident $CD69^{+}CD103^{+}CD8^{+}$T cells. Correlation was assessed using Spearman analysis. Data shown are means ± SEM with significance determined by one-way ANOVA with a Dunn's post hoc test for multiple comparisons. *p<0.05, **p<0.01.

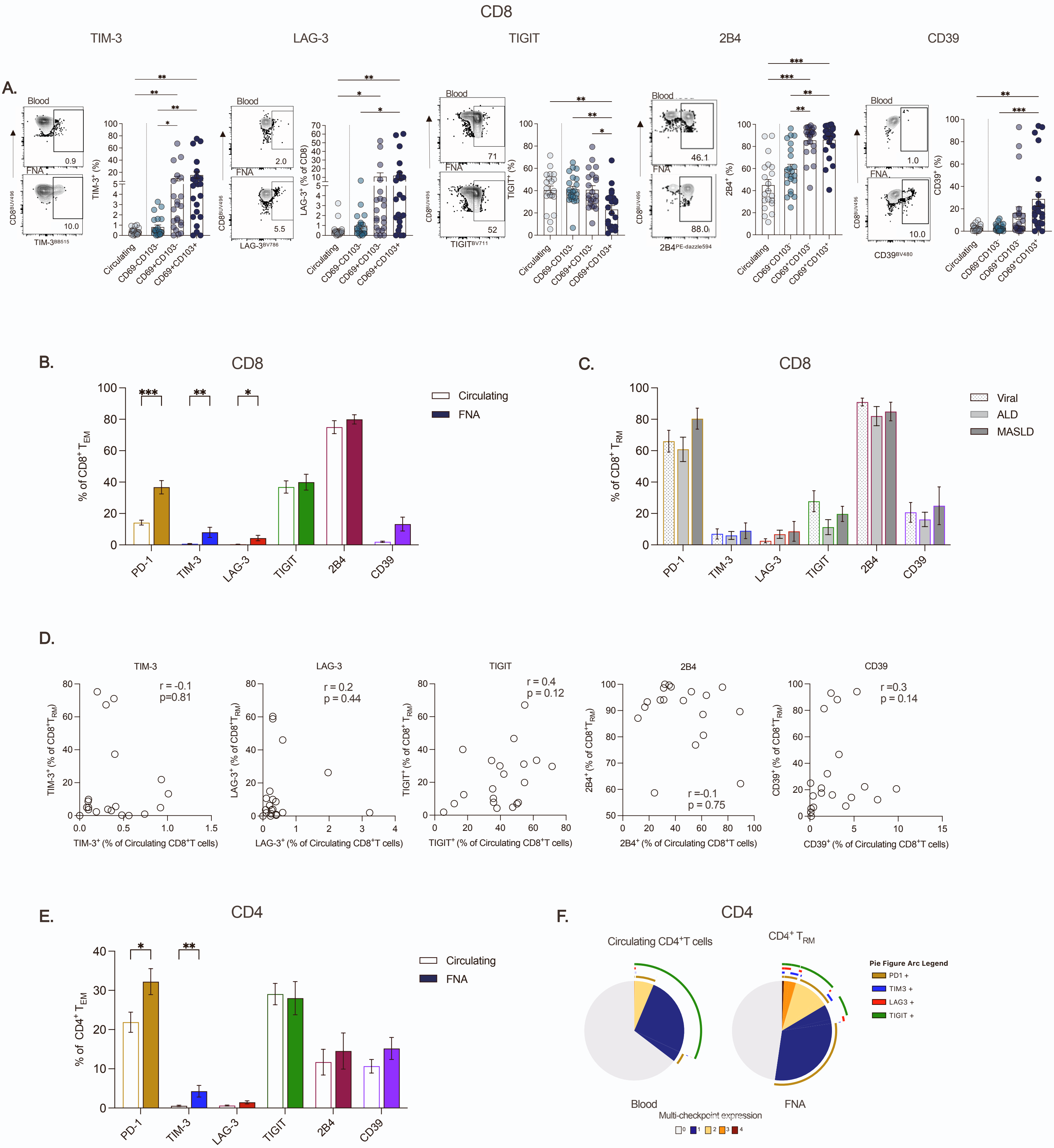

Fig. S4. Enrichment of immune checkpoints in HCC FNA. (A) Representative flow cytometric plots and summary frequency data of CD8$^+$T cells expressing TIM-3, LAG-3, TIGIT, 2B4 and CD39 checkpoint molecules, showing percentage among circulating subsets in blood, tumour recirculating CD69$^-$CD103$^-$, single positive CD69$^+$CD103$^-$ or tissue resident CD69$^+$CD103$^+$ CD8$^+$T$_{RM}$ in FNA. (B) Summary frequency data of

CD8+TEM (CD62L-) subsets expressing the examined checkpoint molecules in blood and FNA (n=22). (C) Summary frequency data of checkpoint molecule expressing subsets within FNA $CD8^{+}TRM$ grouped by underlying HCC aetiology in samples stained for these markers: viral (HCV/HBV, n=6) and non-viral (ALD, n=5, MASLD, n=7). (D) Scatter plots depicting correlation between the frequency of immune checkpoint expressing $CD8^{+}T$ cells within blood $CD8^{+}T$ cells versus FNA $CD8^{+}T_{RM}$. (E) Summary frequency data of $CD4^{+}T_{EM}$ ($CD62L^{-}$) subsets expressing the examined checkpoint molecules in blood and FNA (n=22). (F) SPICE charts showing the proportion of $CD4^{+}T$ cells expressing PD-1, TIM-3, LAG-3 or TIGIT among global circulating versus FNA $CD69^{hi}CD4^{+}T_{RM}$. Coloured slices indicate the number of checkpoints expressed on T_{RM}, while colour of the arc indicates the identity of checkpoint molecule expressed (n=16). Data shown are mean ± SEM and significance were determined by one-way ANOVA with a Dunn's post hoc test for multiple comparisons (A), multiple t-test and non-parametric test (B, E) and non-parametric Spearman correlation(D). *p<0.05; **p<0.01; ***p<0.001,****p<0.0001.
